# Intergenerational metabolic toxicity of perfluorooctanesulfonic acid exposure in adult offspring rats: a multi-omics approach

**DOI:** 10.3389/fendo.2025.1589826

**Published:** 2025-09-18

**Authors:** Guoqi Yu, Tingyu Luo, Xiaona Huo, Xi Meng, Liping Feng, Yan Sun, Yongjie Liu, Jun Zhang

**Affiliations:** ^1^ Ministry of Education-Shanghai Key Laboratory of Children’s Environmental Health, Xinhua Hospital, Shanghai Jiao Tong University School of Medicine, Shanghai, China; ^2^ Global Centre for Asian Women’s Health, Yong Loo Lin School of Medicine, National University of Singapore, Singapore, Singapore; ^3^ Department of Obstetrics & Gynaecology, Yong Loo Lin School of Medicine, National University of Singapore, Singapore, Singapore; ^4^ School of Public Health, Guilin Medical University, Guilin, China; ^5^ Department of Obstetrics, International Peace Maternity and Child Health Hospital, Shanghai, China; ^6^ Department of Obstetrics and Gynecology, Duke University Medical Center, Durham, NC, United States

**Keywords:** perfluorooctane sulfonate, glucose homeostasis, early-life exposure, multi-omics, intergenerational impact

## Abstract

**Introduction:**

Perfluorooctane sulfonate (PFOS), known as a critical endocrine disruptor, was linked to potential intergenerational effect in population studies. Yet, the toxic metabolic mechanisms remain unclear, particularly at relatively low PFOS concentration.

**Methods:**

This study investigated the metabolic impacts of early-life (pregnancy and lactation) PFOS exposure on adult Sprague-Dawley (SD) offspring rats using an integrated transcriptomics and metabolomics approach. Metabolic phenotypes, including glucose tolerance, lipids, and metabolic biomarkers were measured.

**Results:**

Early-life exposure to 0.03 mg/kg PFOS was found to be associated with elevated fasting and 15-minute blood glucose, serum insulin, and adiponectin levels and a decrease of leptin level in dose of 0.3 mg/kg was observed. Differentially expressed genes induced by PFOS exposure were enriched in NOD-like receptor signaling, parathyroid hormone synthesis, secretion and action, unsaturated fatty acid biosynthesis, insulin signaling, retinol metabolism, fatty acid metabolism, glucagon signaling, type II diabetes, and PPAR signaling. Differentially expressed metabolites were linked to citric acid cycle, glycerophospholipid metabolism, and fatty acid biosynthesis. Coenrichment analysis revealed feature changes in several pathways, including glycerophospholipid metabolism, sphingolipid metabolism, and primary bile acid synthesis (0.03 mg/kg), and retinol metabolism, linoleic acid metabolism, DGlutamine and D-Glutamine biosynthesis, and fatty acid elongation (0.3 mg/kg).

**Conclusion:**

Early-life exposure to PFOS might lead to metabolic perturbations in adult offspring, which might be triggered by changes in pathways, i.g. glycerophospholipid metabolism, retinol metabolism, linoleic acid metabolism, and fatty acid elongation. Further validation of these pathways is required.

## 1 Introduction

The Developmental Origins of Health and Disease (DOHaD) theory raised that early life-stage exposure to unfavorable chemical, physical, nutritional, or psychosocial environments may induce long term and diverse health issues. These effects are not limited to hypoplasia, low immunity, growth retardation, and neurobehavioral developmental disorders during the perinatal and childhood stages. Instead, they may extend into adulthood and increase the susceptibility to metabolic disease i.g. obesity, type 2 diabetes, and cardiovascular disease (1–3).

Per- and poly-fluoroalkyl substances (PFAS), as persistent chemicals, are renowned for their unique surfactant qualities, which have led to their widespread utilization in various daily-life products such as non-stick pans, fire extinguishers, and carpets (4). Although the phase-out of PFOS and Perfluorooctanoic Acid (PFOA) in some Western countries, their production unabated in certain Asian countries, thus potentially creating risks for human metabolic well-being due to their pervasive, long-lasting, and bio-accumulative nature (5). Both of them have been detected across tissues such as umbilical cord blood, liver, brain, and adipose tissues, etc. (6, 7). Importantly, the placenta is not an effective barrier to PFOS due to its physicochemical properties—particularly its small molecular size, high protein binding, and resistance to metabolism. As a result, PFOS can cross the placenta and accumulate in the fetus and potentially interfere with critical developmental processes (8). Evidence from epidemiological studies indicated that maternal PFAS exposure can directly affect fetal growth and development via the umbilical cord, thereby resulting in an elevated risk of adverse birth outcomes (9). Additionally, intrauterine PFAS exposure was recognized as having potential detrimental effects such as blood pressure abnormalities and atopic dermatitis in young offspring (10–13). Recent reviews indicate that intrauterine and postnatal PFAS exposure at the early-life stage may influence the offspring’s physical growth, obesity, and the onset of menarche, although findings remain inconsistent (14).

Studies in rodent animals revealed that exposure to PFOS at a dosage of 1.5 mg/kg·bd could result in a notable and statistically significant elevation of metabolic indicators, such as levels of insulin, leptin, the area-under-curve (AUC) value at 10 weeks after weaning, glucose (increased but not significant), and decreased adiponectin level in adult offspring rats (15). Exposure to PFOS at low concentrations during early life has been found to induce long-term elevated blood glucose levels in offspring, alongside the development of dysregulated glucose and insulin resistance (16). In contrast, another animal experiment involving neonatal rats exposed to 20 mg/kg·bd PFOS revealed a notable decrease in glucose levels and changes in blood glucose levels were not observed in adult rats exposed to gradient doses of 0.5-5.0 mg/kg·bd (16). In line with the above-mentioned inconsistent alterations of metabolic phenotypes at different doses among adult rats, intrauterine exposure to PFOS and PFOA did not result in obesity among adult offspring mice, while PFOA exhibited a markable decrease of glucose, specifically in the doses at 0.01 and 0.1 mg/kg·bd (17). Long-term exposure of PFAS mixture chemicals in adult male mice may trigger aberrant sperm methylation and gene expression of offspring liver and fat (18). One study examined the developmental toxicity of PFOS but the outcome endpoints were limited to metabolic traits in newborn rat only (19). Other studies, including ours, have shown significant metabolic perturbances of PFOS and perfluorobutane sulfonate (PFBS) exposure during pregnancy on maternal rats, but their impacts on offspring remain largely unknown (20, 21). Taking together, the majority of previous toxicological investigations only focused on rodents who experienced PFOS exposure during adulthood at high exposed doses or pregnancy toxicity. Long-term metabolic intergenerational effects of intrauterine exposure have long been overlooked, especially when exposure at relatively low level. Thus, studies examining the intergenerational impacts of early-life PFOS exposure are critical, especially for studies with lower exposure concentrations, longer exposure durations, and a more sensitive growth window for animals.

Fortunately, the emergence of multi-omics technologies, including transcriptomics and metabolomics, has revolutionized the field of toxicology. These advanced approaches offer high throughput and sensitivity, enabling the simultaneous detection of multiple targets and identification of low-abundance gene expressions and metabolites. While numerous studies have applied metabolomics in toxicological research, the novelty of our work lies in the integrated application of both transcriptomics and metabolomics to comprehensively and systematically characterize the molecular perturbations induced by PFOS exposure. By combining these two powerful approaches, our study provides deeper insights into the coordinated changes at both the gene and metabolite levels, allowing for more precise identification of disrupted biological pathways and critical molecular targets that may underlie PFOS toxicity.

This study endeavored to: 1) comprehensively assess the intergenerational glucolipid changes of PFOS exposure in adult offspring, and 2) unravel the possible mechanisms through the utilization of multi-omics technique.

## 2 Methods

### 2.1 Animal treatment

The PFOS Potassium Salt, with a purity of 98% and CAS number 2795-39-3, was procured from Toronto Research Chemicals. 3% starch gel was prepared to dissolve PFOS and administered once daily via oral gavage throughout gestation and lactation. The selection of oral gavage doses of FPOS at 0.03 and 0.3 mg/kg body weight was derived from the human tolerable daily intake (TDI): 150 ng/kg body weight and the reference dose (RfD): 20 ng/kg·d launched by the European Food Safety Authority and the Environmental Protection Agency of the United States, respectively. To consider variations between humans and mice, a correction factor of 10× for inter-individual differences and an interspecies correction factor (toxicodynamics: 3 and toxicokinetics: 81) were applied (22, 23). Thus, the relevant doses range of 0.03 and 0.3 mg/kg·bw·d were finally chosen to mimic PFOS exposure faced by ordinary people. The lower dose (0.03 mg/kg·bw·d) may reflect high-end exposure levels in the general population, while the higher dose (0.3 mg/kg·bw·d) is comparable to levels observed in occupational settings. The calculation formulas were described elsewhere (24).

Female and male Sprague-Dawley (SD) rats aged 9-11 weeks were procured from Hunan Silaike Jingda (China). Following a one-week acclimatization period, SD rats were permitted to mate overnight (female: male, 2:1). The verification of pregnancy was confirmed by the presence of a vaginal plug. Subsequently, Random assignment placed pregnant rats into three treatment groups, each consisting of 7-8 rats. From gestational day 1 (GD1) until the time of sacrifice, they were administered the assigned treatment orally. Pregnant maternal rats were accommodated in a controlled environment with specific pathogen-free conditions, ensuring a temperature between 21-25°C, humidity levels maintained at 40-60%, and a 12-hour light-dark photoperiod.

Body weight and diet consumption of maternal rats were diligently documented daily. The postpartum rats were euthanized at postnatal day 35 (PND 35) after they finished lactation (PND 21), and measurements of litter weight, number of fetuses, and stillbirths were recorded both at birth and 3 days post-birth. Weaned offspring rats from all dams of maternal rats were pooled and 10 of each treated group were randomly selected in a 1:1 ratio of females to males (5 females and 5 males) to constitute the F1 generation. After weaning at three weeks of age, the pups were housed individually. Offspring rats per group were fed without any treatment and body weight and dietary consumption every week were recorded until sacrificed in the 12^th^ week. In this study, both female rats (PND 35) and offspring rats (9 weeks) were euthanized through intraperitoneal injection of 8% chloral hydrate under anesthesia, and various tissues, including blood, liver, and pancreas were collected. The Ethics Committee of Guilin Medical University approved this study.

### 2.2 Assessment of metabolic phenotypes

#### 2.2.1 Oral glucose tolerance test and lipid profile

For the OGTT, 12-week-old offspring rats were subjected to an overnight fast (12 hours) (25). Fasting blood glucose was promptly quantified by an automated blood glucose meter (Logitech). Subsequently, the rats were orally administered 50% glucose solution and glucose levels were tested at intervals of 15, 30, 60, and 120 minutes post-gavage, and the areas under the curve (AUC) were calculated to facilitate quantitative comparisons. In this study, AUC was used to reflect the function of insulin secretion and the ability to maintain normal blood glucose levels. An increased AUC suggest either insufficient insulin secretion or insulin resistance, where cells do not secret enough insulin to lower the external glucose level or exhibit reduced responsiveness to insulin, requiring higher insulin levels to control blood glucose.

Serum from fasting aortic blood samples was obtained and subsequently analyzed by an automated biochemical analyzer (XL-600, ERBA, Germany) to quantify the lipid profile, including total triglyceride (TG), total cholesterol (TC), high-density lipoprotein (HDL), and low-density lipoprotein (LDL).

#### 2.2.2 Adipokine measurement

In this study, enzyme-linked immunosorbent assay (ELISA) was applied to assess the serum expression levels of metabolic molecules including adiponectin (CATALOG# 80570), lepti n(CATALOG# 90040), and insulin (CATALOG#H203-1-1). Homeostatic Model Assessment of Insulin Resistance (HOMA-IR) was derived.

### 2.3 Transcriptomic sequencing

From each group, four liver samples (male: female= 2:2) were randomly chosen for transcriptomic sequencing. Total RNA was extracted using the TRIzol reagent (Invitrogen) following the manufacturer’s instructions. RNA integrity and purity were assessed using the Agilent 2100 Bioanalyzer (Agilent Technologies) and NanoDrop spectrophotometer. Only samples with RNA Integrity Number (RIN) ≥ 7.0, OD260/280 between 1.8–2.2, and total RNA >1 µg were included for library preparation. Subsequently, mRNA was isolated and utilized to construct cDNA libraries following the standardized Illumina protocol. Basically, mRNA was enriched using oligo(dT) magnetic beads, fragmented, and reverse-transcribed into cDNA. After end-repair, A-tailing, adapter ligation, and PCR amplification, the libraries were quantified by Qubit and validated by Bioanalyzer. Sequencing was performed using the Illumina HiSeq™ platform, generating ~20–50 million paired-end reads per sample that underwent subsequent processing to acquire clean reads. Raw reads were quality filtered using fastp, removing adapter contamination, low-quality reads (Phred score < 20), and reads with >10% unknown bases (N). Clean reads were aligned to the reference genome using HISAT2. Mapping rates ≥90% were expected. Samples with unusually low mapping rate, high duplication rate, or low total reads (<20 million) were flagged and reviewed. These reads were then aligned to the reference genome sequence. Principal Component Analysis (PCA) and hierarchical clustering were used to detect outlier samples. Outliers due to technical artifacts (e.g., low RNA quality, batch failure) were excluded before downstream analyses.

DESeq and q-values (adjusted p-values by Benjamini and Hochberg method) were utilized to determine the variations in gene expression. Genes with a q-value of less than 0.05 were recognized as showing differentially expressed genes (DEGs). The Kyoto Encyclopedia of Genes and Genomes (KEGG) database and the Gene Ontology (GO) database were employed for gene annotation, biological interpretation, and clustering analysis. Quantitative Real-Time PCR (qRT-PCR) was used to confirm the differentially expressed target genes identified from RNA sequencing. Eight differentially expressed genes and one housekeeping gene were selected. The gene-specific primers were listed in **Supplementary Table S1**. Total RNA was reverse-transcribed using a PrimeScript RT Reagent Kit. qPCR was performed on a CFX96 Real-Time PCR System (Bio-Rad) using SYBR Green Master Mix. Candidate gene selection of housekeepers was pre-evaluated using geNorm and NormFinder to confirm stable expression across conditions. Housekeeping Gene: typically, GAPDH were used. More details were reported elsewhere (24).

### 2.4 Metabolomic profiling

Eight offspring liver samples (male: female= 4:4) in each treated group were chosen randomly and ultrahigh-performance liquid chromatography-mass spectrometry (UPLC-MS) was applied for non-targeted metabolomic analysis, details were reported (24). Progenesis QI software was used for metabolite alignment and quantification. Peak deconvolution was done with default settings. QC samples were injected at regular intervals throughout the run to monitor instrument stability. Features with high missing rates (>20% in QC or >50% in samples) or low repeatability (coefficient of variation >30% in QC samples) were excluded. Signal drift was corrected using QC-based robust LOESS signal correction. Metabolite identification involved a two-step process using generated MS1/MS2 pairs. Initially, an in-house library was employed, which included chemical standards and a meticulously curated compound list featuring precise mass, retention time, and spectral patterns. Metabolomics data were normalized using internal standard-based correction. Following this, data were log-transformed and Pareto-scaled to stabilize variance for downstream analyses. Additional identification methods involved the use of accurate mass, isotope pattern, and MS/MS spectra against public databases such as HMDB, PubChem, METLIN, and KEGG.

### 2.5 Statistical analysis

Differential gene expressions were analyzed using DESeq. PCA was employed to investigate metabolite variability. Furthermore, Orthogonal Partial Least Squares-Discriminant Analysis (OPLS-DA) was utilized to identify differential metabolites by filtering out orthogonal variables unrelated to categorical variables, thus obtaining robust information on group differences. Differential metabolites were confirmed based on the *p*-value< 0.05.

Body weight, organ/body weight ratio, blood glucose levels, lipid levels, and metabolic factors were described as mean ± standard error (SE). Group comparisons were conducted using one-way ANOVA analysis or nonparametric tests where appropriate. LSD test or Dunnett’s T3 test was applied in terms of the homogeneity of variance among different groups. Given that the ratio of male: female rats among each treated group was same and no sex difference was observed, thus no further stratified analysis by offspring gender was conducted.

### 2.6 Multi-omics integration

To benefit a comprehensive interpretation of the biological relationships between DEGs and differentially expressed metabolites (DEMs) in the offspring liver, we performed simultaneous mapping to the KEGG pathway. This integrative analysis aimed to elucidate the potential perturbed biological pathways induced by early-life PFOS exposure. Further, the metabolic pathway analysis for the candidate genes and metabolites was conducted by online tool MetaboAnalyst 5.0.

All data analyses were conducted using R 3.4.1 and SPSS 22.0. A significance level of 0.05 in a two-sided test was used.

## 3 Results

### 3.1 Early-life PFOS exposure and phenotype changes in offspring

Results showed that early-life PFOS exposure resulted in a subsequent reduction of body weight in 2- and 3-week-old offspring rats (**Supplementary Figure S1**). Average body weights of offspring rats at 0.03 and 0.3 mg/kg body weight PFOS exposure was significantly lower than control group. However, catch-up growth was observed from the 4th postpartum week, with a subsequent non-significant change in body weight. Results of living births and 3-day surviving pups were not significant changed across groups.

**Figure 1:**
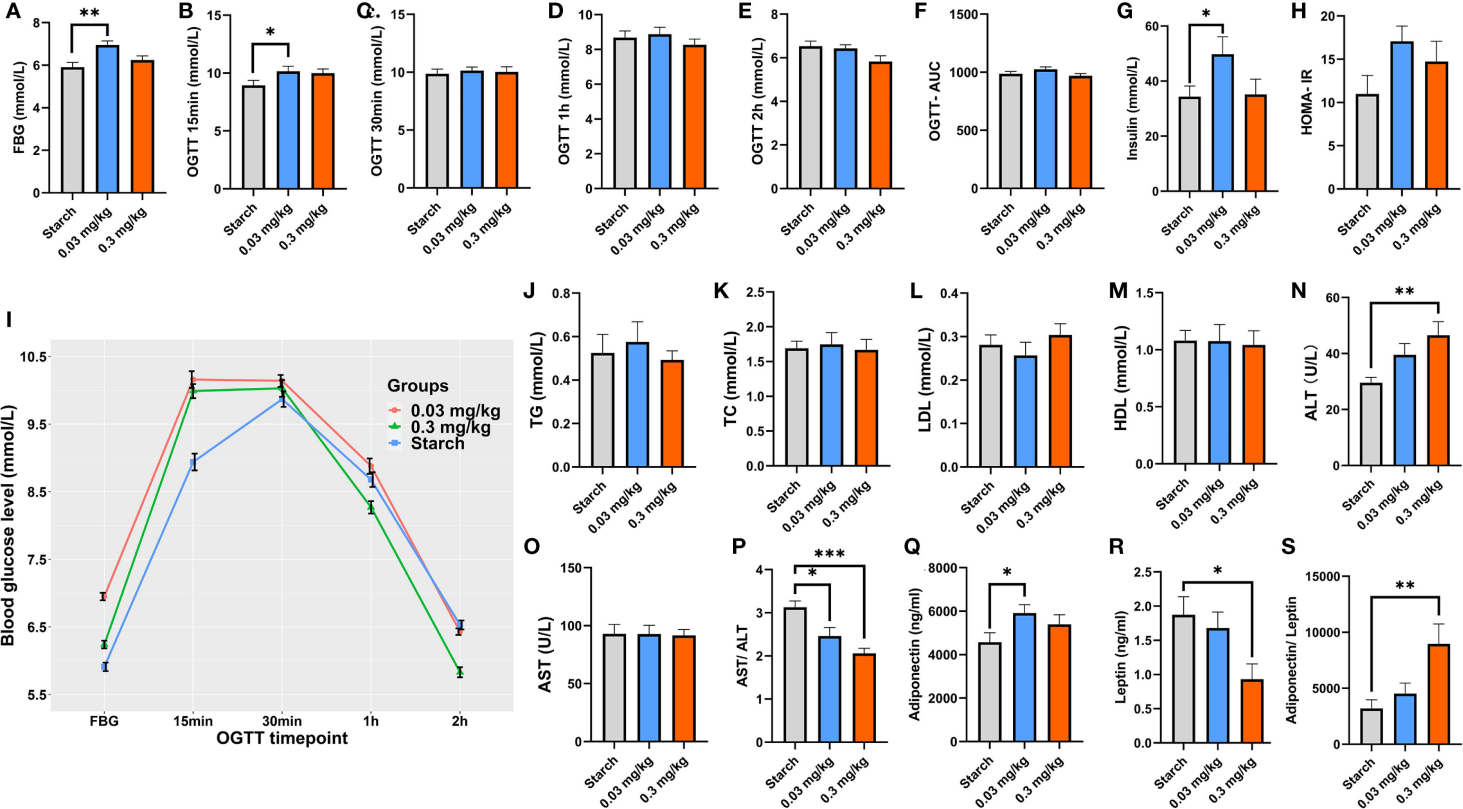
Impact of PFOS exposure during early life on glucose tolerance, lipid profiles, and adipokines of 9-week-old offspring rats. **(A–H, J–S)**: x-axis refer to different exposure groups and y-axis refer to different metabolic indicators. **(I)**: OGTT glucose levels for different exposure groups. Mean and standard error were reported in broken line plot and bar plot. The sample size of control and PFOS treatment at doses of 0.03 and 0.3 mg/kg were 10, 10 and 10, respectively, with male: female ration= 5:5 across groups. A two-tailed p-value< 0.05 was designated as the significance threshold.*p*-value< 0.05 was designated as the significance threshold.

Analysis of OGTT revealed a correlation of PFOS exposure with increased blood glucose levels during fasting and at 15 minutes in adult offspring rats, as illustrated in **Figure 1**. PFOS exposure also showed a trend towards increased AUC values, though not reaching statistical significance (0.03 mg/kg.bd PFOS group vs control group: 1025.18 vs 987). Moreover, significant elevation in insulin levels (*p* < 0.05) was observed in 0.03 mg/kg group (49.8 vs 34.4 mmol/L in control group). Although not statistically significant, there was a tendency noted for an elevation in HOMA-IR within the PFOS-treated groups. Early-life PFOS exposure was linked to increased alanine transaminase (ALT) levels and decreased aspartate transaminase (AST)/ALT ratio. However, no significant changes were found in the lipid profile. Analysis of metabolic factors revealed that exposure to 0.03 mg/kg PFOS during early life significantly increased serum adiponectin levels (5914 vs 4576 ng/ml in control group), while exposure to 0.3 mg/kg PFOS significantly decreased leptin levels (1.87 vs 0.93 ng/ml in control group) and adiponectin/leptin ratio, compared to the controls (8965 vs 3189) (*p*< 0.01).

Evaluation of liver pathology indicated the absence of hemorrhage, edema, or inflammatory cell infiltration in the liver, as well as no evidence of hepatocyte steatosis or ballooning (**Supplementary Figure S2**). Similarly, the pancreas pathology assessment revealed no signs of islet cell swelling, inflammatory cell infiltration, or vacuolization. Furthermore, no significant changes were found in the relative weights of the liver and pancreas in the PFOS exposure groups.

### 3.2 Alterations in the transcriptomic sequencing

**Figure 2:**
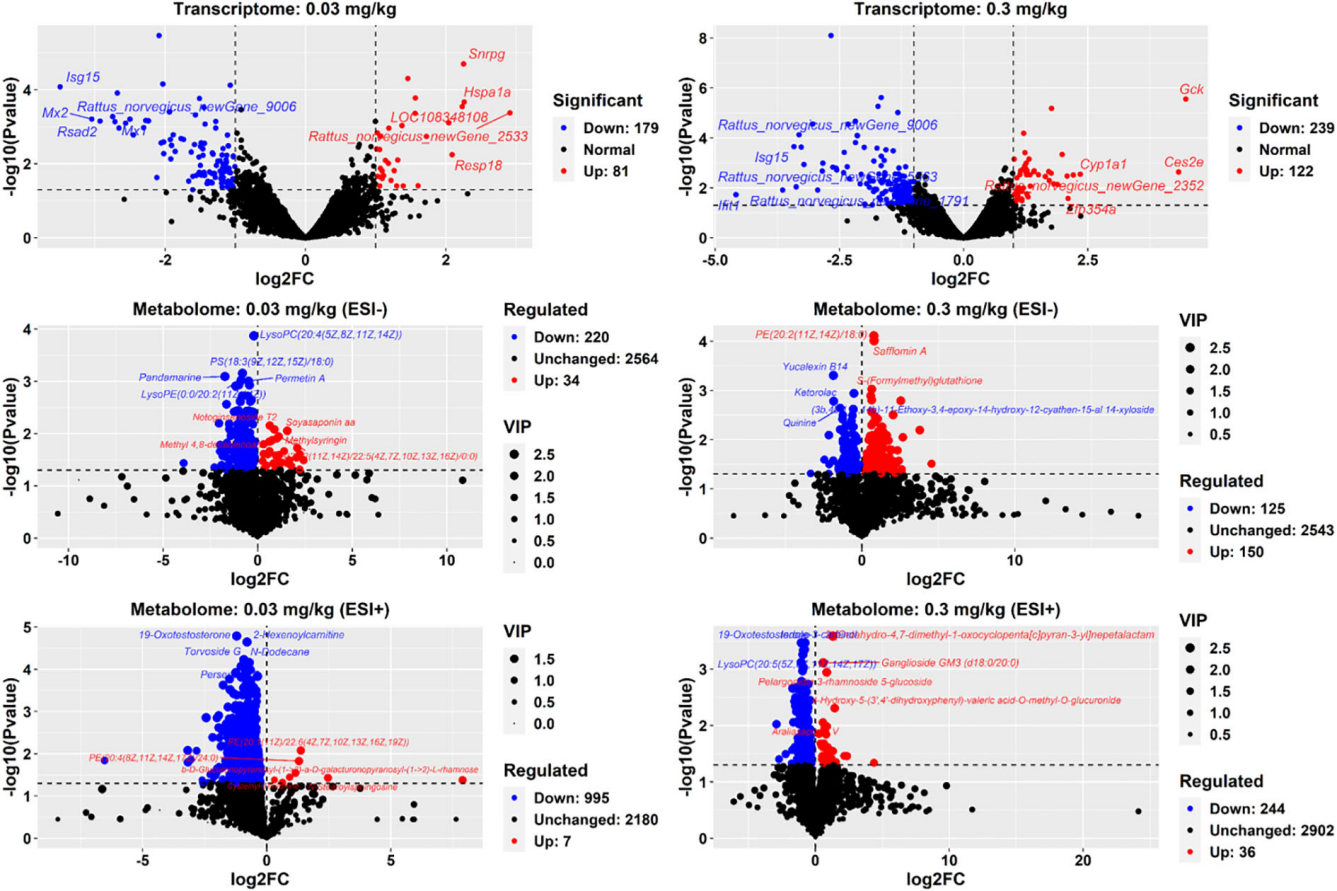
DEGs and DEMs in 9-week-old offspring rats. DEGs: differentially expressed genes. DEMs: differentially expressed metabolites. Each transcriptome sequencing group comprised 4 liver samples (male: female ration= 2:2). A cutoff of *p*-value<0.05 was employed to determine up and down regulation. For metabolome measurements, 8 liver samples (male: female ration= 4:4) were used in each group with a cutoff of *p*-value<0.05 and log2FC>0 or <0 applied. In the volcano plot, red points indicate up-regulated gene expression or metabolites, while blue points signify down-regulation. The names of the top 5 significantly up or down-regulated genes and metabolites were annotated.

260 DEGs in the liver of adult offspring rats exposed to 0.03 mg/kg PFOS were identified, with 81 up-regulated and 179 down-regulated genes (**Figure 2**). Similarly, in the 0.3 mg/kg PFOS group, 361 DEGs were identified, comprising 122 up-regulated and 239 down-regulated genes.

**Figure 3:**
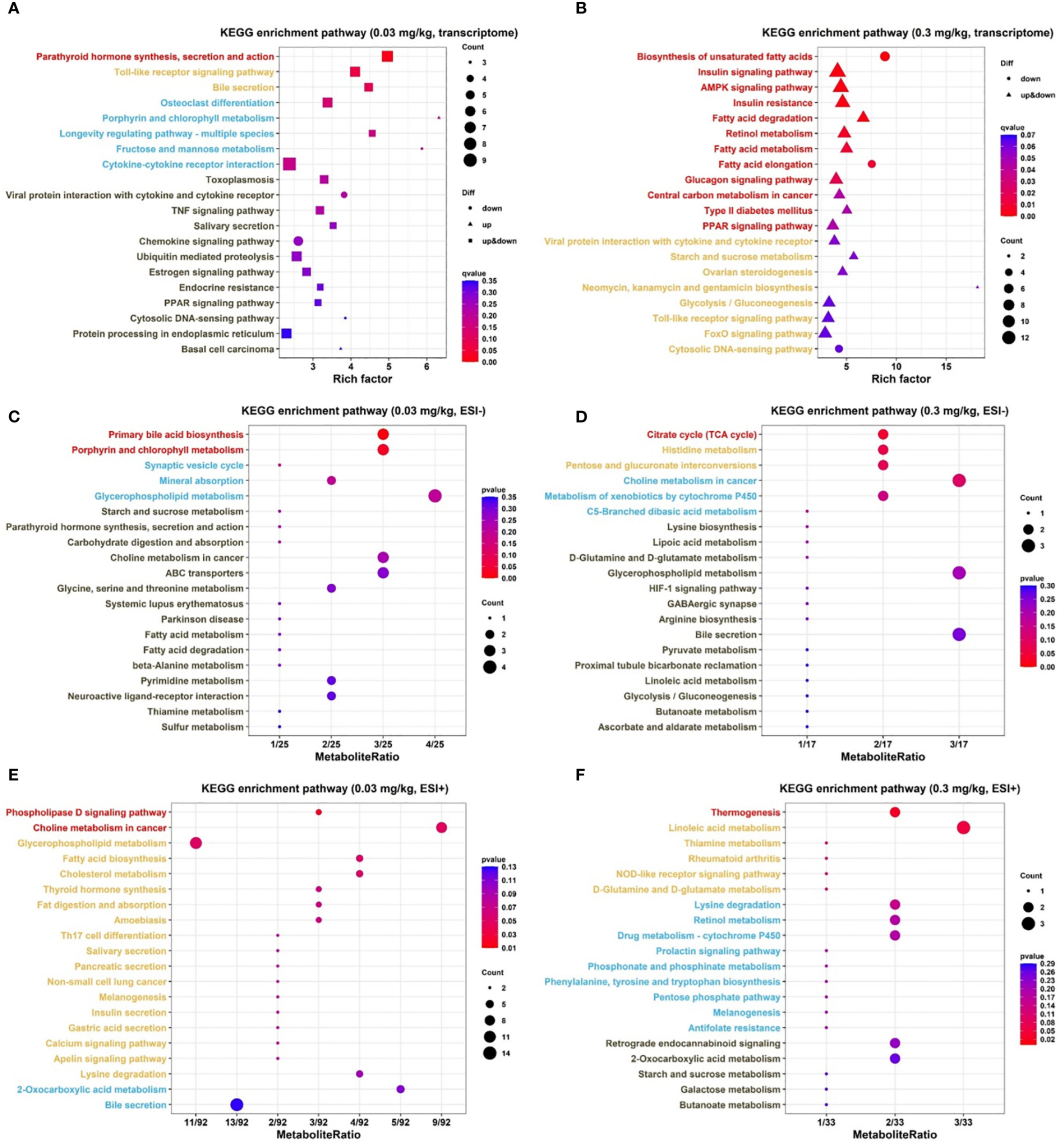
KEGG pathway analysis of DEGs and DEMs (N=4 for each group of transcriptomics and N=8 for each group of metabolomics) in 9-week-old offspring rats. **(A, B)**: KEGG enrichment pathways for transcriptome of different exposure groups. **(C, D)**: KEGG enrichment pathways for metabolome (-) of different exposure groups. **(E, F)**: KEGG enrichment pathways for metabolome (+) of different exposure groups. DEGs: differentially expressed genes. DEMs: differentially expressed metabolites. Each symbol represents a KEGG pathway. On the y-axis, pathway names were indicated and Y-axis text labels were color-coded to indicate their corresponding significance levels based on q-value or p-value: red (<0.05), yellow (0.05-0.10), blue (0.10-0.20), and black (0.20-). The top 20 KEGG enriched pathways were visualized.

Pathway enrichment analysis demonstrated that PFOS exposure at 0.03 mg/kg resulted in significant pathway changes in the parathyroid hormone synthesis, secretion and action pathway, as depicted in **Figure 3A**. Moreover, 0.3 mg/kg dose led to changes in multiple pathways, including biosynthesis of unsaturated fatty acids, insulin signaling pathway, AMPK signaling pathway, insulin resistance, fatty acid degradation, retinol metabolism, fatty acid metabolism, fatty acid elongation, glucagon signaling pathway, type II diabetes mellitus, and PPAR signaling pathway (**Figure 3B**). Detailed information regarding the enrichment analysis can be found in **Supplementary Table S2**.

To validate the findings, qPCR analysis was conducted, and the results of all examined genes in qPCR analysis demonstrated a consistent expression pattern with the transcriptomic data (**Supplementary Figure S3**).

### 3.3 Alterations in the metabolomic profile

Non-targeted UPLC-MS analysis of adult offspring liver generated 3350 ion fragments. The OPLS-DA model was utilized for analyzing metabolite differences between groups (**Figure 2**; **Supplementary Figure S4**). Notably, a total of 254 metabolites (34 increased and 220 decreased, negative ion mode) exhibited significant alterations in the 0.03 mg/kg dosed group, including the up-regulated metabolites, such as top 5 most significantly increased metabolites: PS (14:1(9Z)/16:0), PE(15:0/20:1(11Z)), fasciculic acid C, PE(18:4(6Z,9Z,12Z,15Z)/18:0), mesobilirubinogen, and beta-Elemonic acid, and down-regulated metabolites: notoginsenoside T1, (3S,3’R,5R,6R)-7’,8’-Didehydro-3,6-epoxy-5,6-dihydro-beta, beta-carotene-3’,5-diol, carbetocin, and tigecycline. Meanwhile, 1002 metabolites (7 increased and 995 decreased, positive ion mode) were altered in the 0.03 mg/kg dosed group. For the 0.3 mg/kg dosed exposure, 275 metabolites were changed, including 150 increased metabolites (such as isolimonic acid, homocarnosine, fasciculi acid C, tanacetol A, ligustroside, and O2’-4a-cyclic-tetrahydrobiopterin) and 125 decreased metabolites (such as 2-Hexaprenyl-3-methyl-6-methoxy-1,4 benzoquinone, colistin, dicyclomine, and fumonisin B4) with the highest |log2FC| and VIP values. Similarly, 280 metabolites (positive ion mode) were observed to be changed in the 0.3 mg/kg PFOS group, with 36 increased and 244 decreased metabolites, respectively.

Classification results from the HMDB database indicated that the differentiated metabolites due to PFOS exposure at dose of 0.03 mg/kg (negative ion mode) were primarily associated with glycerophospholipids, prenol lipids, fatty acyls, carboxylic acids and derivatives, steroids and steroid derivatives, organooxygen compounds, and imidazolopyrimidines (**Supplementary Figure S5**). Similarly, the altered metabolites at dose of 0.3 mg/kg exhibited similarities with the 0.03 mg/kg group and were mainly involved in glycerophospholipids, carboxylic acids and derivatives, organooxygen compounds, fatty acyls, steroids and steroid derivatives. The enriched metabolic pathways of the differentiated metabolites at both PFOS doses (positive ion mode) were largely consistent with those in the negative ion mode.

Further annotation and enrichment analysis of the DEMs using the KEGG database revealed metabolic pathway changes at dose of 0.03 mg/kg, primarily involving the altered pathways of primary bile acid biosynthesis and porphyrin and chlorophyll metabolism in the negative ion mode, as well as the phospholipase D signaling pathway and choline metabolism in cancer in the positive ion mode (**Figures 3C, E**). Besides, PFOS exposure in 0.3 mg/kg group mainly affected the citrate cycle pathway (negative ion mode) and thermogenesis (positive ion mode) (**Figures 3D, F**). Detailed statistics of the metabolites enriched in each significant pathway were summarized in **Supplementary Table S3**.

### 3.4 Integrated multi-omics analysis

**Figure 4:**
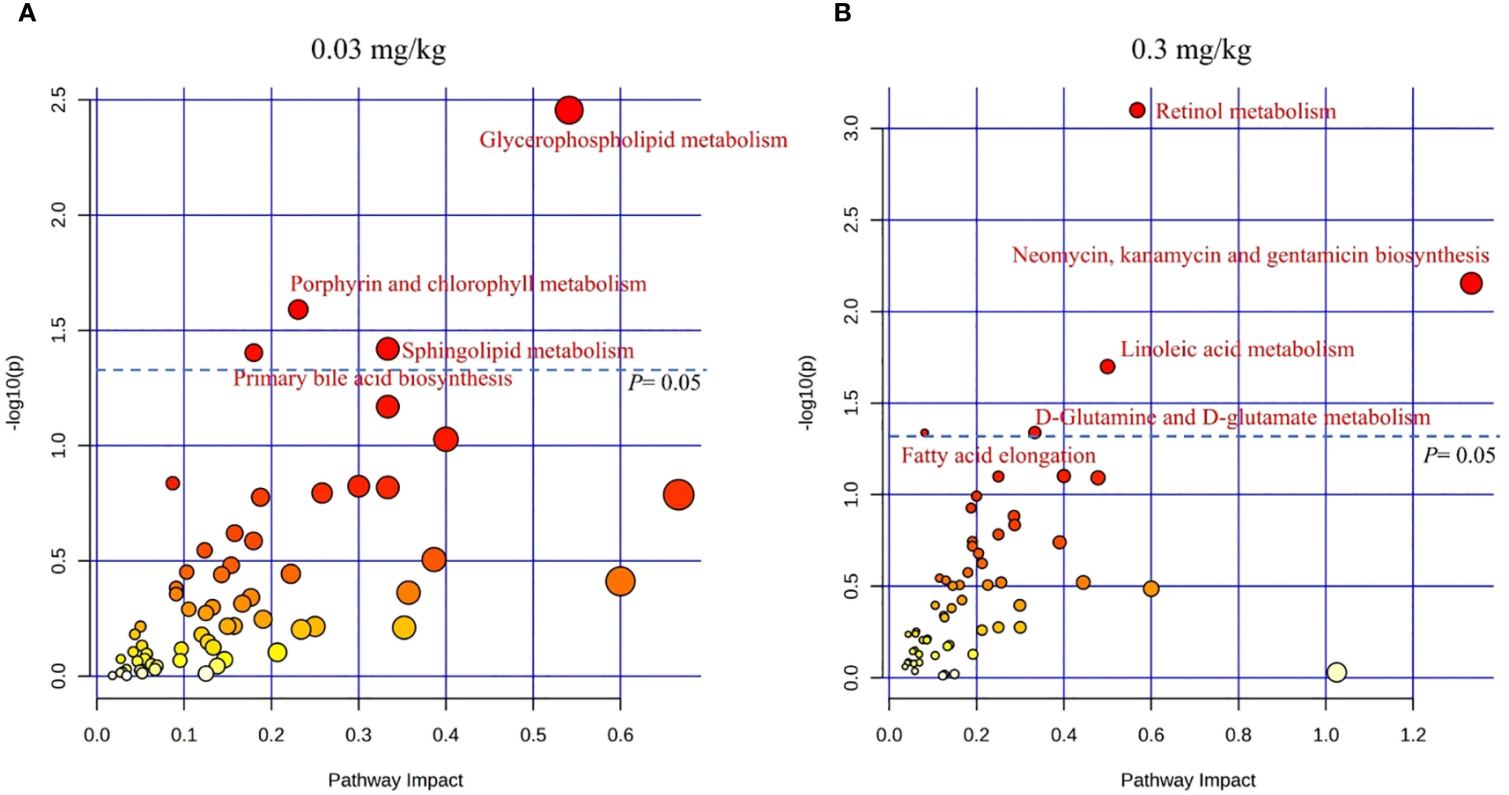
KEGG co-enrichment analysis for DEGs and DEMs in livers of 9-week-old offspring rats (N=4 for each group in transcriptomics and N=8 for each group in metabolomics). **(A)** Co-enrichment analysis for 0.03 mg/kg PFOS exposed group vs control group; **(B)** Co-enrichment analysis for 0.3 mg/kg PFOS exposed group vs control group. DEGs, differentially expressed genes. DEMs, differentially expressed metabolites. Significant pathways were marked as red font, and the color of the circle indicates significance, with a deeper shade of red corresponding to lower *p*-values.

An in-depth analysis of the DEGs and DEMs was applied using MetaboAnalyst 5.0. The impact-value threshold was derived through pathway topology analysis, and the top 10 metabolic pathways with the lowest *p*-values were shown as the most potential target pathways (**Figure 4**). These included glycerophospholipid metabolism, porphyrin and chlorophyll metabolism, sphingolipid metabolism, primary bile acid biosynthesis, alpha-Linolenic acid metabolism, glutathione metabolism, biosynthesis of unsaturated fatty acids, one-carbon pool by folate, taurine and hypotaurine metabolism, and pentose and glucuronate interconversions. In 0.03 mg/kg PFOS exposed group, four pathways exhibited significance, namely glycerophospholipid metabolism, porphyrin and chlorophyll metabolism, sphingolipid metabolism, and primary bile acid biosynthesis. In the case of 0.3 mg/kg PFOS exposure, five out of the ten pathways were significant, including retinol metabolism, neomycin, kanamycin and gentamicin biosynthesis, linoleic acid metabolism, D-Glutamine and D-glutamate metabolism, and fatty acid elongation. Detailed statistics of the co-enriched pathway analysis and the genes and metabolites involved in each pathway were summarized in **Supplementary Table S4**.

**Figure 5:**
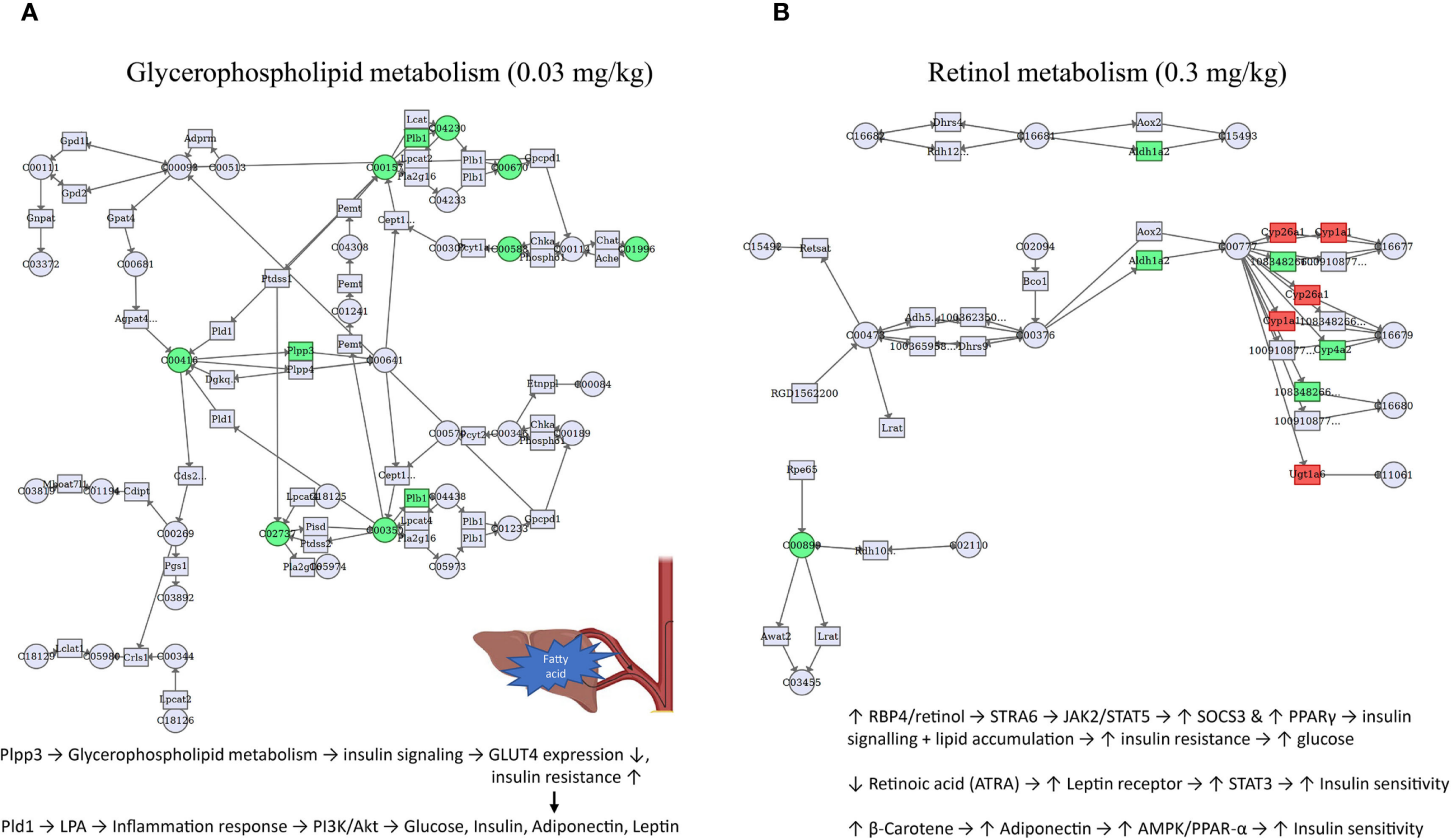
Co-enriched pathways of DEGs and DEMs induced by early-life PFOS exposure in 9-week-old offspring rats (N=4 for transcriptomics and N=8 for metabolomics). **(A)** pathway of glycerophospholipid metabolism for 0.03 mg/kg group. **(B)** pathway of retinol metabolism for 0.3 mg/kg group. DEGs, differentially expressed genes. DEMs, differentially expressed metabolites. Only the most significant pathways of each dose of PFOS exposure group were presented. Official name of genes and metabolites, information of other significant pathways can be referred to **Supplementary Tables S4, S5**. The filled color accounts for the direction of gene or metabolite regulation (red: up-regulated, green: down-regulated).

To enhance the understanding of molecular connectivity and toxicity mechanisms related to differentially expressed genes and differential metabolites, these features were projected onto the co-enrichment pathways of the different PFOS exposure groups. The most significant co-enrichment pathways and proposed mechanistic hypothesis were depicted in **Figure 5**, and the annotated names, statistics of the group comparisons and feature predictions were summarized in **Supplementary Table S5**. At 0.03 mg/kg PFOS exposure, glycerophospholipid metabolism emerged as the most significantly perturbed pathway, while at 0.3 mg/kg, retinol metabolism was prominently affected. These dose-dependent pathway alterations suggest differential mechanisms of metabolic disruption.

## 4 Discussion

The intrauterine and early childhood periods represent crucial stages of fetal growth and development, characterized by heightened susceptibility to environmental chemical exposures. Building upon previous epidemiological findings, this study aimed to establish an early-life exposure model in SD rats to unravel the detrimental effects of PFOS exposure during early development on the metabolic health of adult offspring. Our findings revealed significant disruptions in glucose homeostasis and alterations in adipokine profiles induced by early-life PFOS exposure. Through comprehensive analysis, we identified specific metabolic pathways, as well as corresponding genes and metabolites, that were perturbed by PFOS exposure. These mechanistic insights contribute to a deeper understanding of the metabolic toxicity induced by PFOS exposure and pave the way for further environmental regulation of PFOS.

### 4.1 Metabolic phenotype

Previous studies showed dose-dependent reproductive toxicity of PFOS, with significant effects observed at higher doses. For example, a dose of 3.2 mg/kg·d of PFOS during pregnancy was found to significantly reduce the number of live births, prolong the pregnancy duration, and decrease the number of blastocysts. However, no significant embryotoxicity was observed in other exposure groups (0, 0.1, 0.4, 1.6 mg/kg·d) (26). Similarly, a dose of 1.5 mg/kg·d of PFOS during pregnancy led to a significant weight loss of offspring, while an exposure of 3.0 mg/kg·d resulted in a significant increase in neonatal offspring deaths (27). In this study, we used relatively low doses based on environmental limit standards. We observed that PFOS exposure tend to lower the body weight of offspring, but a catch-up growth occurred from the fourth week. This finding aligns with a population-based study showing an association of prenatal PFAS exposure with lower standardized BMI in infants, with a weakened association as age advances (28, 29).

This study found that 0.3 mg/kg·d PFOS exposure could increase FBG and OGTT 15min blood glucose levels in the offspring adult rats. FBG testing serves as a convenient screening method for diabetes (30). Although no significant differences were observed in blood glucose levels during OGTT at 1h and 2h, and AUC values, the elevated FBG and OGTT 15min glucose levels indicated an imbalance in glucose homeostasis in the PFOS-L group (0.03 mg/kg·d), suggesting early damage to glucose tolerance. Additionally, the fasting insulin level was increased in the 0.3 mg/kg·d PFOS group, indicating early insulin resistance. Interestingly, our study did not observe a dose-response trend between PFOS and glucose changes in offspring rats. Previous studies had reported severe liver and kidney damage, liver cell congestion, necrosis, and inflammatory cell infiltration with high PFOS exposure, leading to a complete disruption of blood glucose homeostasis and even rat mortality (31). Wang et al. demonstrated that high PFOS exposure (30 mg/kg·d) led to a significant decrease in blood glucose levels, while exposure doses of 0.3 and 3.0 mg/kg·d resulted in increased blood glucose levels (32). Perinatal exposure to PFOS at doses of 0.3 and 3.0 mg/kg·d induced elevated serum insulin levels, increased insulin resistance index, and impaired β-cell function in adult offspring rats (16). Notably, the doses of 0.3 and 3.0 mg/kg·d used in previous studies were approximately 10 times and 100 times higher than the equivalent exposure dose in general population, with the higher dose (3.0 mg/kg·d) reflecting occupational exposure levels (33). Our study extended the investigation to a lower PFOS exposure level of 0.03 mg/kg·d, which approximates the human equivalent exposure dose. The results indicated that PFOS may potentially affect blood glucose homeostasis at this exposure level. Nevertheless, this study did not reveal a dose-response relationship due to the utilization of lower exposure doses, shorter exposure times, and a limited sample size, the observed subtle phenotypic alterations in the PFOS-treated groups, coupled with alterations in gene expression and metabolites, still hold considerable significance. This is particularly noteworthy given the exposure levels employed in this study.

Adiponectin, a specific protein secreted by adipocytes, binds to G protein-coupled receptors and plays pivotal role in enhancing insulin sensitivity, regulating carbohydrate and lipid metabolism, and correcting hyperinsulinemia and insulin resistance (34). Consistent with our findings of increased serum adiponectin levels in offspring rats exposed to 0.03 mg/kg·d PFOS, studies conducted in the Taiwan Hypertensive Adolescent cohort and the Viva cohort demonstrated that exposure to PFNA and PFHxS, respectively, also increased serum adiponectin levels (35, 36).

Leptin, an adipokine produced by adipocytes, acts in opposition to adiponectin and is crucial in the development of insulin resistance, obesity, and diabetes. The adiponectin/leptin ratio is a sensitive indicator for assessing the risk of insulin resistance, and cardiovascular diseases (37). Interestingly, our study revealed a significant decrease in leptin levels and increase in the adiponectin/leptin ratio following exposure to 0.3 mg/kg·d PFOS, which contrasts with several previous studies reporting increased leptin levels and decreased adiponectin levels associated with PFAS exposure (38, 39). However, Shelly et al. demonstrated a significant inverse association between PFOS, PFDA, and reduced leptin levels in girls aged 7 and 13 years, which can support our findings (40). Additionally, In a study conducted within the Viva cohort, no significant correlation was observed between PFAS and levels of adiponectin or leptin (41), suggesting that the roles of adiponectin and leptin in PFAS-induced metabolic abnormalities remain inconclusive. It is worth noting that even though individuals with typical diabetes or lipid abnormalities often exhibit significant reductions in adiponectin and elevations in leptin, early compensatory mechanisms may happen in generally healthy individuals or animal models. Furthermore, the health effects of metabolic factors such as leptin may follow a “U” shaped pattern, where high levels can lead to leptin resistance and obesity, while low levels may be linked to immune dysfunction and infection (42, 43).

### 4.2 Altered gene expression and relevant pathways

Transcriptomic sequencing analysis using liver samples from adult offspring rats exposed to PFOS during early life revealed significant metabolic alterations primarily associated with parathyroid hormone (PTH) pathways and multiple pathways related to fatty acid metabolism, glycemic control, and insulin resistance. PTH is a vital hormone involved in maintaining calcium homeostasis and bone mineralization. Dysregulated PTH secretion can lead to conditions such as primary or secondary hyperparathyroidism, often accompanied by renal diseases. Elevated PTH levels may also impact cardiovascular health and the development of diabetes mellitus (44, 45). As the relationship between PFAS exposure and PTH remains unclear, further investigation is warrant to unveil the underlying mechanisms of action.

As observed in this study, the effects of PFOS on pathways related to glucolipid metabolism involve several genes with significantly changed expression levels, such as *Acot*, *Elovl*, *Irs2*, *G6pc*, *Gck*, *Gys2*, *Pik3r1* (*Pi3ka*), *Socs3*, *Ppp1r3c*, *Ppp1r3b*, *Fasn*, *Foxo1*, *Cpt1a*, *Ldha*. *Acot1*, *Acot2*, *Acot4*, *Acot3*, *Elovl2* and *Elovl5* are among the key genes that affect lipid and sterol metabolism. Among them, *Acyl coenzyme A thioesterases* (*Acot*) are an important class of peroxisomes that catalyze the synthesis of free fatty acids and coenzyme A from acyl coenzyme A (short, medium, long and extra-long chains), bile acid-CoA and methyl-branched-CoA (46). *Elovl2* and *Elovl5* are important regulatory genes for polyunsaturated fatty acids and *Elovl2* prolongs the synthesis of arachidonic acid, eicosapentaenoic acid *Elovl2* lengthens the carbon chain length of arachidonic, eicosapentaenoic, docosapentaenoic and linolenic acids and also contributes to the synthesis of PPARα ligands. Similarly, *Elovl5* is involved in the elongation of a variety of polyunsaturated long-chain fatty acids from C18-C20 (47). *Irs2*, *G6pc*, *Gck*, *Gys2*, and *Pik3r1* are important regulatory genes in the tricarboxylic acid cycle and insulin signaling pathway (48). In addition to the classical attenuated negative feedback regulation of cytokine signaling, *Socs* have been increasingly suggested to play an critical role in receptor tyrosine kinase signaling, such as inhibition of insulin signaling. the *Socs* protein family has effects in inhibiting leptin and insulin signaling pathways. Evidence suggests that *Socs3* plays critical role in the leptin resistance, with Socs proteins (Socs1, Socs3, Socs6 and Socs7) can reduce insulin action (49). *Ppp1r3c* and *Ppp1r3b* can be widely expressed in liver, muscle, and cardiac muscle and can target glycogen granules by dephosphorylating and activating glycogen synthase Ppp1r3c heterozygous deletion in mice resulting in reduced tissue glycogen levels with progressive glucose intolerance, hyperinsulinemia and insulin resistance with age (50). Fatty acid synthase (Fasn) is an important regulatory enzyme in adipose ab initio synthesis, while abnormal expression of *Fasn* is closely associated with the development of diabetes (51). Foxo1 serves as a critical transcription factor in glucose metabolism. In the liver, *Foxo1* becomes active during fasting and is deactivated upon feeding in typical physiological circumstances. This process is one of the crucial mechanisms through which insulin can rapidly and effectively inhibits hepatic glucose production during the postprandial state. In cases of insulin resistance, phosphorylated Foxo1 translocates into the nucleus, where it associates with PGC-1α, thereby triggering the expression of target genes accounted for gluconeogenesis in the liver, such as *G6p* and *Pepck* (52). Cpt1 serves as a pivotal factor regulating the intake of long-chain fatty acids into mitochondria for β-oxidation. Situated on the outer mitochondrial membrane, Cpt1 transforms long-chain acyl-coenzyme A into acylcarnitine equivalents. These equivalents are subsequently transported into the mitochondria by acylcarnitine translocase, only to be converted back into acyl-coenzyme A by *Cpt2* (53). Cpt1a is one of the three isoforms of Cpt1, and Cpt1a deficiency can cause acute fatty liver and hepatic encephalopathy during pregnancy (54). Lactate dehydrogenase a (Ldha) is one of the key rate-limiting enzymes in anaerobic enzymes of the body and is an important factor in maintaining glucose homeostasis.

### 4.3 Metabolomic alterations and metabolic pathways

In this study, metabolomics analysis of livers in offspring adult rats revealed that PFOS exposure induced both porphyrin and chlorophyll metabolism, citrate cycle, and disturbances in lipid metabolism, involving mainly malic acid, α-ketoglutarate, glucuronide bilirubin, bile acid, glycine, levulinic acid, phenylalanine, L-urobilin, mesobilirubinogen, bilirubinogen, S-acetyldihydrothioctinamide-E, taurine. In addition to malic acid, extended carboxylic acid and levulinic acid have an impact on metabolic process of offspring rats (55, 56). α-ketoglutaric acid is also an vital intermediate in the tricarboxylic acid cycle, while glucuronide bilirubin, bile acid, L-urea bilirubin, mesobilirubinogen, and bilirubinogen are mainly important metabolites or raw materials in bile acid metabolism, suggesting that PFOS exposure may trigger disturbances in bile acid metabolism (57). Perturbation of bile acid synthesis from cholesterol may be one of the pathways through which PFAS affects blood and liver cholesterol levels. Results from animal studies suggest that PFOS and PFOA exposure increases serum bile acid levels and decreases bile acid secreted in the feces, while causing a downregulation of *CYP7A1* expression and consequently an imbalance in cholesterol homeostasis (58, 59). In addition, CYP7A1 is regulated by FXR, but HNF4α and PPARα also play a regulatory role (60). Taurine is a functional amino acid with high content in the organism and has complex biological roles, including anti-inflammatory, antioxidant, and regulation of intracellular calcium ion homeostasis (61). Taurine is an essential part of cellular life processes as well as other differentially altered amino acids such as glycine, phenylalanine, etc. Che-Jung Chang et al. found PFNA exposure to be associated with altered amino acids such as taurine and glycine in a study of PFAS exposure, metabolome of the placenta of pregnant women and fetal growth, which could support the findings of this study (62).

The observation that different PFOS doses perturb distinct metabolic pathways—such as bile acid biosynthesis at 0.03 mg/kg and retinol metabolism and fatty acid elongation at 0.3 mg/kg—likely reflects non-linear, threshold-dependent toxicity mechanisms. PFOS, as a persistent environmental contaminant, is known to exhibit pleiotropic biological effects, and its toxicity often depends not only on dose but also on tissue accumulation, receptor binding affinity, and compensatory metabolic capacity. At lower doses (e.g., 0.03 mg/kg), PFOS may selectively engage high-affinity targets or signaling pathways, such as those regulating bile acid synthesis via FXR (farnesoid X receptor) or related nuclear receptors, leading to subtle but biologically meaningful metabolic disruptions (63). In contrast, higher doses (e.g., 0.3 mg/kg) may overwhelm homeostatic mechanisms, leading to broader metabolic perturbations such as disruptions in retinol metabolism and fatty acid elongation, which may be secondary to mitochondrial stress, ER dysfunction, or PPARα activation (64).

This dose-dependent switching of pathways suggests the existence of multiple toxicity mechanisms that are activated in a hierarchical manner, depending on PFOS exposure levels. It also supports the concept of dose thresholds, where specific toxic effects only emerge beyond certain concentrations. While our study does not allow for the precise determination of these thresholds, the distinct pathway profiles observed at different doses highlight the importance of considering non-linear dose-response relationships in PFOS risk assessment.

### 4.4 Integrative multi-omics

Several co-enriched pathways induced by early-life PFOS exposure were identified in this study. In a recent systematic review paper, changes in glycerophospholipid metabolism and linoleic acid metabolism induced by PFAS exposure were found in several studies, and are essential for vital biological membrane functions, while fatty acids and carnitines play a significant role in the energy supply pathway of fatty acid oxidation (65). For example, perfluorononanoic acid (PFNA) and perfluoroundecanoic acid (PFUnDA) were found to be significantly associated with glycerophosphocholines and linoleic acid metabolism (66). Another case-control study identified 35 PFAS- and type 2 diabetes-related metabolite features and two pathways dominated by glycerophospholipids and diacylglycerols, which can support our findings (67). Altered glycerophospholipid metabolism may impair insulin signaling via Plpp3 downregulation, reducing GLUT4 expression and promoting insulin resistance. In parallel, increased LPA (lysophosphatidic acid) from Pld1 may activate inflammatory pathways that disrupt PI3K/Akt signaling, contributing to dysregulation of glucose homeostasis, leptin, and adiponectin (68).

Besides, significant changes in retinol metabolism was observed in 0.3 mg/kg group, which is consistent with a maternal-child paired study as they found that PFAS exposure in maternal blood was correlated with retinol metabolism (69). Disruption in retinol metabolism may involve elevated RBP4/retinol, activating STRA6–JAK2–STAT5 signaling, which increases SOCS3 and PPARγ, further suppressing insulin signaling and promoting lipid accumulation. Conversely, retinoic acid (ATRA) and β-carotene appear to engage pathways (e.g., STAT3, AMPK/PPAR-α) that may partially counterbalance these effects by improving insulin sensitivity and adiponectin levels (70). More studies are warranted for other co-enriched pathways.

Several limitations merit consideration. Firstly, the non-targeted metabolomics approach used in this study might not have fully encompassed all metabolite varieties due to its inherent characteristics. The constraints of metabolite database might have led to the omission of specific metabolites from distinct pathways. Furthermore, a less stringent significance threshold was applied to determine differential metabolites and pathway enrichment. While this cut-off is commonly used to maximize the identification of differential metabolites for enrichment, it is important to consider the potential inflation of type I errors resulting from multiple testing. Secondly, differential metabolites and biological pathways identified in this study warrant further validation. More detailed metabolic measurements such as Insulin Tolerance Tests (ITT) need to be considered in future studies. Thirdly, omics analysis was limited to liver tissue. While the liver plays a central role in metabolic regulation, contributions from other organs such as adipose tissue, skeletal muscle, and pancreas—particularly in relation to insulin resistance and adipokine secretion—were not directly assessed and warrant further investigation in future multi-tissue studies. Lastly, although this study has identified alterations at the gene expression and metabolic levels associated with early-life PFOS exposure, it remains possible that some phenotypic manifestations of disease outcomes—such as metabolic syndrome, insulin resistance, or obesity—may not fully emerge until later in life or during aging. Therefore, long-term, life-course investigations in offspring following early-life exposure are essential, particularly at environmentally relevant low-dose exposures that may produce subtle but persistent effects. Additionally, the role of epigenetic modifications—such as DNA methylation or histone remodeling—in key metabolic pathway genes warrants further investigation, as these changes may serve as long-lasting “molecular memories” that maintain metabolic dysregulation long after the initial exposure (71). Understanding these epigenetic mechanisms may provide critical insights into how early-life environmental insults can program chronic disease risk (72).

In summary, findings in this study underscore the potential of early-life PFOS exposure to contribute to the development of metabolic disorders such as insulin resistance and type 2 diabetes later in life. They also highlight the importance of incorporating developmental exposure and low-dose effects into human health risk assessments. The results call for strengthened regulatory oversight and further research to better understand and mitigate the intergenerational impacts of PFOS and related environmental contaminants.

## 5 Conclusion

Our study suggested that early-life PFOS exposure has the potential to affect glucose homeostasis in adult offspring rats, as indicated by elevated blood glucose level of OGTT and insulin level, but not a dose-response relationship. Several key co-enriched biological pathways, including glycerophospholipid metabolism and retinol metabolism, as well as DEGs and DEMs, were identified. Findings in this study provide valuable understanding into the fundamental biological mechanism that may contribute to the observed epidemiological associations. However, further *in vitro* and *in vivo* studies are necessary to validate and elucidate the exact toxic pathways involved.

## Data Availability

The raw data supporting the conclusions of this article will be made available by the authors, without undue reservation.
