# Supplementary material for: Intergenerational metabolic toxicity of perfluorooctanesulfonic acid exposure in adult offspring rats: a multi-omics approach
Source: Front Endocrinol (Lausanne). 2025 Sep 18;16:1589826. doi: 10.3389/fendo.2025.1589826 (PMC12488417; doi:10.3389/fendo.2025.1589826)
Supplement: Supplementary file 1 [file DataSheet1.docx]

**Table S1. The qPCR primer sequences for the validation of transcriptomic results.**

| Gene name | Forward | Reverse |
| --- | --- | --- |
| Gapdh | TATGACTCTACCCACGGCAAG | ATACTCAGCACCAGCATCACC |
| Zbtb16 | CGCCACCTTCGCTCACATACAG | ACTTCTTGCCACAGCCATTACACTC |
| Hhex | AGTACCATTCTTCTAGCGTTT | ACGAAGTCACATTTAGCCACT |
| Gcnt2 | TCCATCCATCCATCCATCCATTTGC | CTGTCTTGCTTCCCTCATGTCTGTC |
| Fos | CGTCTTCCTTTGTCTTCACCTACCC | TTCTCTGACTGCTCACAGGGCTAG |
| Hdc | TCTACCTCAGACATGCGAAC | GGACCGAATCACAAACCAC |
| Gck | AGGGCATCCTCCTCAATTGGAC | TGGCCACTGTGTCGTTCACC |
| Nceh1 | TGGAAACTGATGCTGCTGGATGC | CGCCGACTACTGTGAACTGATGTG |
| Cyp4a1 | CTCCGTGCTTGGTCTGCTTCTG | GAGGTGATGGGAACTGCTGGAAAG |

**Table S2.** **KEGG pathway analysis of DEGs between PFOS exposure and control groups for 9-week-old offspring rats.**

| Group | Description | Enrich factor | *p*-value | *q*-value | Genes |
| --- | --- | --- | --- | --- | --- |
| 0.03 mg/kg | Parathyroid hormone synthesis, secretion and action | 4.96 | 0.001 | 0.017 | *Cdkn1a;Adcy3;Nr4a2;Pde4b;Fos;Egr1;Pde4d* |
| 0.3 mg/kg | Biosynthesis of unsaturated fatty acids | 8.85 | <0.001 | 0.001 | *Acot2;Scd;Elovl2;Acot4;Acot3;Acot1;Rattus_norvegicus_newGene_8285* |
| 0.3 mg/kg | Insulin signaling pathway | 4.1 | <0.001 | 0.002 | *Socs3;Ppp1r3b;Foxo1;Ppp1r3g;Ppp1r3c;Pik3r1;Irs2;Hk3;Fasn;G6pc;Gys2;Gck* |
| 0.3 mg/kg | AMPK signaling pathway | 4.43 | <0.001 | 0.002 | *Foxo1;Scd;Cpt1a;Hmgcr;Pik3r1;Pfkfb3;Ppp2r3a;Irs2;Fasn;G6pc;Gys2* |
| 0.3 mg/kg | Insulin resistance | 4.61 | <0.001 | 0.002 | *Socs3;Ppp1r3b;Foxo1;Cpt1a;Ppargc1b;Ppp1r3c;Pik3r1;Irs2;G6pc;Gys2* |
| 0.3 mg/kg | Fatty acid degradation | 6.68 | <0.001 | 0.002 | *Eci1;Cyp4a1;Cyp4a3;Cpt1a;Acsl3;Acat2;Rattus_norvegicus_newGene_8285* |
| 0.3 mg/kg | Retinol metabolism | 4.78 | <0.001 | 0.006 | *Ugt2a3;Cyp4a1;Cyp4a3;Cyp26b1;Aldh1a1;Cyp1a1;Cyp2s1;LOC100361547* |
| 0.3 mg/kg | Fatty acid elongation | 7.55 | <0.001 | 0.009 | *Acot2;Elovl2;Acot4;Acot3;Acot1* |
| 0.3 mg/kg | Fatty acid metabolism | 5.01 | <0.001 | 0.009 | *Scd;Cpt1a;Elovl2;Acsl3;Acat2;Fasn;Rattus_norvegicus_newGene_8285* |
| 0.3 mg/kg | Glucagon signaling pathway | 3.96 | 0.001 | 0.017 | *Sik1;Ldha;Foxo1;Cpt1a;G6pc;Gys2;Gck;Rattus_norvegicus_newGene_6535* |
| 0.3 mg/kg | Central carbon metabolism in cancer | 4.29 | 0.003 | 0.044 | *Ldha;Fgfr3;Pik3r1;Hk3;Gck;Rattus_norvegicus_newGene_6535* |
| 0.3 mg/kg | Type II diabetes mellitus | 5.04 | 0.003 | 0.046 | *Socs3;Pik3r1;Irs2;Hk3;Gck* |
| 0.3 mg/kg | PPAR signaling pathway | 3.63 | 0.003 | 0.046 | *Cyp4a1;Cyp4a3;Scd;Cpt1a;Acsl3;Cyp8b1;Rattus_norvegicus_newGene_8285* |
| 0.3 mg/kg | Ovarian steroidogenesis | 4.61 | 0.004 | 0.057 | *Acot2;Cyp1a1;Acot4;Acot3;Acot1* |
| 0.3 mg/kg | Neomycin, kanamycin and gentamicin biosynthesis | 18.13 | 0.005 | 0.057 | *Hk3;Gck* |
| 0.3 mg/kg | Viral protein interaction with cytokine and cytokine receptor | 3.79 | 0.005 | 0.057 | *Xcr1;Ccl4;Ackr4;Csf1;Cxcl9;Cxcl10* |
| 0.3 mg/kg | Starch and sucrose metabolism | 5.72 | 0.005 | 0.057 | *Hk3;G6pc;Gys2;Gck* |
| 0.3 mg/kg | Glycolysis / Gluconeogenesis | 3.25 | 0.006 | 0.063 | *Ldha;Hk3;AABR07025010.1;G6pc;Gck;Rattus_norvegicus_newGene_1307;Rattus_norvegicus_newGene_6535* |
| 0.3 mg/kg | Cytosolic DNA-sensing pathway | 4.25 | 0.006 | 0.063 | *Il1b;Zbp1;Ccl4;Irf7;Cxcl10* |
| 0.3 mg/kg | Toll-like receptor signaling pathway | 3.17 | 0.007 | 0.063 | *Il1b;Ccl4;S100a4;Irf7;Pik3r1;Cxcl9;Cxcl10* |
| 0.3 mg/kg | FoxO signaling pathway | 2.86 | 0.007 | 0.063 | *Bcl6;Rasgef1b;Gadd45g;Foxo1;Plk3;Pik3r1;Irs2;G6pc* |

Notes: DEGs: differentially expressed genes. Only KEGG pathways with q-value <0.05 were listed.

**Table S3. KEGG pathway analysis of DEMs between PFOS exposure and control groups for 9-week-old offspring rats.**

| Group | Ion model | Description | Enrich factor | *P*-value | *Q*-value | Metabolites |
| --- | --- | --- | --- | --- | --- | --- |
| 0.03 mg/kg | Negative | Primary bile acid biosynthesis | 6.14 | 0.008 | 0.375 | Cholic acid; Taurine; Glycine |
| 0.03 mg/kg | Negative | Porphyrin and chlorophyll metabolism | 4.09 | 0.029 | 0.667 | Mesobilirubinogen; Glycine; Bilirubin glucuronide |
| 0.03 mg/kg | Positive | Phospholipase D signaling pathway | 3.63 | 0.020 | 0.606 | Sphingosine 1-phosphate; DG(16:0/18:2(9Z,12Z)/0:0); Prostaglandin F2a |
| 0.03 mg/kg | Positive | Choline metabolism in cancer | 1.72 | 0.047 | 0.606 | Phosphorylcholine; LysoPC(20:0); LysoPC(16:0); LysoPC(22:4(7Z,10Z,13Z,16Z)); LysoPC(22:5(7Z,10Z,13Z,16Z,19Z)); DG(16:0/18:2(9Z,12Z)/0:0); LysoPC(20:4(5Z,8Z,11Z,14Z)); Glycerophosphocholine; LysoPC(22:5(4Z,7Z,10Z,13Z,16Z)) |
| 0.3 mg/kg | Negative | Citrate cycle (TCA cycle) | 6.02 | 0.038 | 0.505 | alpha-ketoglutarate; S-Acetyldihydrolipoamide-E |
| 0.3 mg/kg | Positive | Thermogenesis | 6.75 | 0.027 | 0.619 | L-Carnitine; Anandamide |
| 0.3 mg/kg | Positive | Linoleic acid metabolism | 3.37 | 0.049 | 0.619 | 9,12,13-TriHOME; 9,10-DHOME; 9(S)-HPODE |

Notes: DEGs: differentially expressed genes. DEMs: differentially expressed metabolites. Only KEGG pathways with *p*-value <0.05 were listed.

**Table S4. Summary statistics for KEGG co-enrichment analysis.**

| Dosed groups | Co-enriched KEGG Pathways | Total | Expected | Hits | Raw p-value | FDR | Impact | Matched_features |
| --- | --- | --- | --- | --- | --- | --- | --- | --- |
| 0.03 mg/kg | Glycerophospholipid metabolism | 86 | 3.75 | 10 | 0.004 | 0.29 | 0.54 | cpd:C00350; cpd:C00157; cpd:C04230; cpd:C00588; cpd:C01996; cpd:C00416; cpd:C02737; cpd:C00670; rno:298579; rno:313977 |
| 0.03 mg/kg | Porphyrin and chlorophyll metabolism | 53 | 2.31 | 6 | 0.026 | 0.83 | 0.23 | cpd:C00037; cpd:C01079; cpd:C03263; cpd:C05770; rno:65155; rno:289533 |
| 0.03 mg/kg | Sphingolipid metabolism | 58 | 2.53 | 6 | 0.038 | 0.83 | 0.33 | cpd:C06124; cpd:C01120; cpd:C00550; cpd:C06126; cpd:C01190; rno:313339 |
| 0.03 mg/kg | Primary bile acid biosynthesis | 90 | 3.92 | 8 | 0.04 | 0.83 | 0.18 | cpd:C00037; cpd:C00245; cpd:C01301; cpd:C00695; cpd:C05452; cpd:C17337; cpd:C05122; rno:25428 |
| 0.03 mg/kg | alpha-Linolenic acid metabolism | 22 | 0.96 | 3 | 0.068 | 1 | 0.33 | cpd:C00157; cpd:C06427; rno:298579 |
| 0.03 mg/kg | Glutathione metabolism | 56 | 2.44 | 5 | 0.094 | 1 | 0.4 | cpd:C00051; cpd:C00127; cpd:C00037; cpd:C00077; rno:362196 |
| 0.03 mg/kg | Biosynthesis of unsaturated fatty acids | 47 | 2.05 | 4 | 0.146 | 1 | 0.09 | cpd:C00249; cpd:C06429; cpd:C06428; cpd:C06427 |
| 0.03 mg/kg | One carbon pool by folate | 31 | 1.35 | 3 | 0.15 | 1 | 0.3 | cpd:C00440; cpd:C00664; rno:362657 |
| 0.03 mg/kg | Taurine and hypotaurine metabolism | 16 | 0.7 | 2 | 0.152 | 1 | 0.33 | cpd:C00245; cpd:C05122 |
| 0.03 mg/kg | Pentose and glucuronate interconversions | 32 | 1.39 | 3 | 0.161 | 1 | 0.26 | cpd:C03033; cpd:C02266; rno:289533 |
| 0.3 mg/kg | Retinol metabolism | 45 | 1.58 | 7 | 0.001 | 0.07 | 0.57 | cpd:C00899; rno:24188; rno:312495; rno:24296; rno:308445; rno:50549; rno:289533 |
| 0.3 mg/kg | Neomycin, kanamycin and gentamicin biosynthesis | 4 | 0.14 | 2 | 0.007 | 0.29 | 1.33 | rno:25060; rno:24385 |
| 0.3 mg/kg | Linoleic acid metabolism | 17 | 0.6 | 3 | 0.02 | 0.56 | 0.5 | cpd:C00157; cpd:C14826; rno:298579 |
| 0.3 mg/kg | D-Glutamine and D-glutamate metabolism | 10 | 0.35 | 2 | 0.046 | 0.77 | 0.33 | cpd:C02237; cpd:C00026 |
| 0.3 mg/kg | Fatty acid elongation | 75 | 2.64 | 6 | 0.046 | 0.77 | 0.08 | cpd:C01944; rno:192272; rno:314304; rno:50559; rno:681337; rno:498728 |
| 0.3 mg/kg | Glycerophospholipid metabolism | 86 | 3.03 | 6 | 0.079 | 0.85 | 0.4 | cpd:C00350; cpd:C00157; cpd:C04230; cpd:C00346; rno:298579; rno:362219 |
| 0.3 mg/kg | Butanoate metabolism | 29 | 1.02 | 3 | 0.08 | 0.85 | 0.25 | cpd:C00026; rno:65984; rno:308100 |
| 0.3 mg/kg | Biosynthesis of unsaturated fatty acids | 47 | 1.65 | 4 | 0.081 | 0.85 | 0.48 | rno:192272; rno:314304; rno:50559; rno:681337 |
| 0.3 mg/kg | Cysteine and methionine metabolism | 71 | 2.5 | 5 | 0.102 | 0.95 | 0.2 | cpd:C04188; cpd:C00155; cpd:C01005; rno:100912604; rno:24533 |
| 0.3 mg/kg | Ubiquinone and other terpenoid-quinone biosynthesis | 17 | 0.6 | 2 | 0.118 | 0.99 | 0.19 | cpd:C00082; cpd:C00828 |

Notes: only KEGG pathways with top 10 lowest p-values of co-enriched pathways in each PFOS exposed groups VS control group were listed.

**Table S5. Key metabolites and genes involving in the significant pathways of KEGG co-enrichment analysis.**

| Dosed groups | KEGG Pathways | KEGG ID/ gene ID | Metabolite/gene | log2FC | Pvalue | P.adjust | VIP | AUC |
| --- | --- | --- | --- | --- | --- | --- | --- | --- |
| 0.03 mg/kg | Glycerophospholipid metabolism | C00157 | PC(24:0/22:5(7Z,10Z,13Z,16Z,19Z)) | -0.59 | 0.01 | 0.11 | 1.53 | 0.83 |
| 0.03 mg/kg | Glycerophospholipid metabolism | C00157 | PC(14:1(9Z)/20:2(11Z,14Z)) | 0.31 | 0.04 | 0.14 | 0.86 | 0.14 |
| 0.03 mg/kg | Glycerophospholipid metabolism | C00157 | PC(22:4(7Z,10Z,13Z,16Z)/P-18:0) | -1.34 | 0.01 | 0.09 | 1.33 | 0.94 |
| 0.03 mg/kg | Glycerophospholipid metabolism | C00157 | PC(20:5(5Z,8Z,11Z,14Z,17Z)/P-16:0) | -0.53 | 0.02 | 0.48 | 1.91 | 0.78 |
| 0.03 mg/kg | Glycerophospholipid metabolism | C00157 | PC(18:4(6Z,9Z,12Z,15Z)/P-16:0) | -1.49 | 0.01 | 0.48 | 2.22 | 0.97 |
| 0.03 mg/kg | Glycerophospholipid metabolism | C00157 | PC(18:0/20:3(5Z,8Z,11Z)) | -0.66 | 0.01 | 0.48 | 2.06 | 0.88 |
| 0.03 mg/kg | Glycerophospholipid metabolism | C00157 | PC(20:3(5Z,8Z,11Z)/P-18:0) | -1.12 | 0.004 | 0.47 | 2.38 | 0.95 |
| 0.03 mg/kg | Glycerophospholipid metabolism | C00350 | PE(14:1(9Z)/24:1(15Z)) | -0.82 | 0.03 | 0.13 | 1.26 | 0.78 |
| 0.03 mg/kg | Glycerophospholipid metabolism | C00350 | PE(14:0/20:2(11Z,14Z)) | -1.51 | 0.001 | 0.07 | 1.58 | 0.89 |
| 0.03 mg/kg | Glycerophospholipid metabolism | C00350 | PE(20:4(5Z,8Z,11Z,14Z)/14:1(9Z)) | -0.9 | 0.02 | 0.13 | 1.3 | 0.81 |
| 0.03 mg/kg | Glycerophospholipid metabolism | C00350 | PE(20:1(11Z)/22:6(4Z,7Z,10Z,13Z,16Z,19Z)) | 1.37 | 0.01 | 0.11 | 1.43 | 0.06 |
| 0.03 mg/kg | Glycerophospholipid metabolism | C00350 | PE(22:5(7Z,10Z,13Z,16Z,19Z)/24:0) | -2.19 | 0.05 | 0.15 | 1.00 | 0.80 |
| 0.03 mg/kg | Glycerophospholipid metabolism | C00350 | PE(22:2(13Z,16Z)/P-18:1(11Z)) | -1.31 | 0.04 | 0.14 | 1.11 | 0.70 |
| 0.03 mg/kg | Glycerophospholipid metabolism | C00350 | PE(18:4(6Z,9Z,12Z,15Z)/20:4(5Z,8Z,11Z,14Z)) | -0.77 | 0.02 | 0.12 | 1.23 | 0.83 |
| 0.03 mg/kg | Glycerophospholipid metabolism | C00350 | PE(24:0/24:0) | -1.26 | 0.04 | 0.14 | 1.09 | 0.75 |
| 0.03 mg/kg | Glycerophospholipid metabolism | C00350 | PE(22:2(13Z,16Z)/22:6(4Z,7Z,10Z,13Z,16Z,19Z)) | -1.33 | 0.01 | 0.11 | 1.34 | 0.86 |
| 0.03 mg/kg | Glycerophospholipid metabolism | C00350 | PE(20:4(8Z,11Z,14Z,17Z)/24:0) | 1.3 | 0.01 | 0.12 | 1.45 | 0.16 |
| 0.03 mg/kg | Glycerophospholipid metabolism | C00350 | PE(22:0/P-18:1(11Z)) | -1.08 | 0.0007 | 0.06 | 1.60 | 0.97 |
| 0.03 mg/kg | Glycerophospholipid metabolism | C00350 | PE(18:1(11Z)/18:0) | 0.53 | 0.02 | 0.50 | 1.95 | 0.22 |
| 0.03 mg/kg | Glycerophospholipid metabolism | C00350 | PE(20:5(5Z,8Z,11Z,14Z,17Z)/16:0) | 0.47 | 0.04 | 0.53 | 1.65 | 0.19 |
| 0.03 mg/kg | Glycerophospholipid metabolism | C00350 | PE(18:3(9Z,12Z,15Z)/22:1(13Z)) | -0.71 | 0.03 | 0.51 | 1.84 | 0.83 |
| 0.03 mg/kg | Glycerophospholipid metabolism | C00350 | PE(18:4(6Z,9Z,12Z,15Z)/18:0) | 2.09 | 0.02 | 0.48 | 2.03 | 0.05 |
| 0.03 mg/kg | Glycerophospholipid metabolism | C00350 | PE(18:3(9Z,12Z,15Z)/20:1(11Z)) | 0.97 | 0.05 | 0.54 | 1.77 | 0.28 |
| 0.03 mg/kg | Glycerophospholipid metabolism | C00350 | PE(18:3(9Z,12Z,15Z)/20:0) | 1.67 | 0.03 | 0.51 | 1.91 | 0.25 |
| 0.03 mg/kg | Glycerophospholipid metabolism | C00350 | PE(15:0/20:1(11Z)) | 2.22 | 0.05 | 0.54 | 1.78 | 0.25 |
| 0.03 mg/kg | Glycerophospholipid metabolism | C00350 | PE(20:3(5Z,8Z,11Z)/P-16:0) | -0.69 | 0.03 | 0.50 | 1.87 | 0.78 |
| 0.03 mg/kg | Glycerophospholipid metabolism | C00416 | LPA(0:0/18:0) | -0.61 | 0.02 | 0.12 | 1.50 | 0.91 |
| 0.03 mg/kg | Glycerophospholipid metabolism | C00416 | LPA(18:1(9Z)/0:0) | -0.47 | 0.004 | 0.09 | 1.45 | 0.89 |
| 0.03 mg/kg | Glycerophospholipid metabolism | C00588 | Phosphorylcholine | -0.47 | 0.002 | 0.07 | 1.71 | 0.92 |
| 0.03 mg/kg | Glycerophospholipid metabolism | C00670 | Glycerophosphocholine | -0.46 | 0.003 | 0.08 | 1.59 | 0.91 |
| 0.03 mg/kg | Glycerophospholipid metabolism | C01996 | Acetylcholine | -0.74 | 0.02 | 0.12 | 1.26 | 0.78 |
| 0.03 mg/kg | Glycerophospholipid metabolism | C02737 | PS(18:0/20:4(8Z,11Z,14Z,17Z)) | -0.83 | 0.01 | 0.10 | 1.51 | 0.86 |
| 0.03 mg/kg | Glycerophospholipid metabolism | C02737 | PS(18:0/20:0) | 0.61 | 0.01 | 0.48 | 2.1 | 0.16 |
| 0.03 mg/kg | Glycerophospholipid metabolism | C04230 | LysoPC(18:2(9Z,12Z)) | -0.54 | 0.0003 | 0.04 | 1.76 | 0.95 |
| 0.03 mg/kg | Glycerophospholipid metabolism | C04230 | LysoPC(18:1(9Z)) | -0.55 | 0.003 | 0.08 | 1.61 | 0.92 |
| 0.03 mg/kg | Glycerophospholipid metabolism | C04230 | LysoPC(22:5(7Z,10Z,13Z,16Z,19Z)) | -0.38 | 0.02 | 0.13 | 1.23 | 0.83 |
| 0.03 mg/kg | Glycerophospholipid metabolism | C04230 | LysoPC(20:4(5Z,8Z,11Z,14Z)) | -0.48 | 0.01 | 0.10 | 1.41 | 0.84 |
| 0.03 mg/kg | Glycerophospholipid metabolism | C04230 | LysoPC(22:5(4Z,7Z,10Z,13Z,16Z)) | -0.49 | 0.01 | 0.11 | 1.28 | 0.86 |
| 0.03 mg/kg | Glycerophospholipid metabolism | C04230 | LysoPC(20:0) | -0.75 | 0.02 | 0.12 | 1.29 | 0.84 |
| 0.03 mg/kg | Glycerophospholipid metabolism | C04230 | LysoPC(16:0) | -0.49 | 0.05 | 0.15 | 1.19 | 0.81 |
| 0.03 mg/kg | Glycerophospholipid metabolism | C04230 | LysoPC(22:4(7Z,10Z,13Z,16Z)) | -0.63 | 0.02 | 0.12 | 1.42 | 0.89 |
| 0.03 mg/kg | Glycerophospholipid metabolism | C04230 | 1-Linoleoylglycerophosphocholine | -1.19 | 0.01 | 0.10 | 1.4 | 0.89 |
| 0.03 mg/kg | Glycerophospholipid metabolism | C04230 | 1-Stearoyl-2-hydroxy-sn-glycero-3-phosphocholine | -0.35 | 0.04 | 0.52 | 1.79 | 0.88 |
| 0.03 mg/kg | Porphyrin and chlorophyll metabolism | C01079 | Protoporphyrinogen IX | -0.71 | 0.04 | 0.14 | 1.17 | 0.73 |
| 0.03 mg/kg | Porphyrin and chlorophyll metabolism | C03263 | Coproporphyrinogen III | -0.55 | 0.02 | 0.12 | 1.23 | 0.83 |
| 0.03 mg/kg | Porphyrin and chlorophyll metabolism | C05770 | Coproporphyrin III | -1.3 | 0.003 | 0.08 | 1.50 | 0.89 |
| 0.03 mg/kg | Porphyrin and chlorophyll metabolism | C00037 | Glycine | -0.34 | 0.04 | 0.53 | 1.72 | 0.78 |
| 0.03 mg/kg | Sphingolipid metabolism | C01190 | Glucosylceramide (d18:1/12:0) | -0.96 | 0.02 | 0.12 | 1.30 | 0.83 |
| 0.03 mg/kg | Sphingolipid metabolism | C06126 | Galabiosylceramide (d18:1/26:0) | -1.25 | 0.05 | 0.15 | 1.08 | 0.78 |
| 0.03 mg/kg | Sphingolipid metabolism | C06126 | Galabiosylceramide (d18:1/26:1(17Z)) | -2.44 | 0.001 | 0.07 | 1.83 | 0.92 |
| 0.03 mg/kg | Sphingolipid metabolism | C01120 | Sphinganine 1-phosphate | -1.15 | 0.01 | 0.09 | 1.52 | 0.91 |
| 0.03 mg/kg | Sphingolipid metabolism | C06124 | Sphingosine 1-phosphate | -0.87 | 0.01 | 0.10 | 1.46 | 0.88 |
| 0.03 mg/kg | Sphingolipid metabolism | C00550 | 2beta-Hydroxytestosterone | -0.71 | 0.02 | 0.12 | 1.31 | 0.89 |
| 0.03 mg/kg | Sphingolipid metabolism | C06126 | Galabiosylceramide (d18:1/24:1(15Z)) | -1.02 | 0.01 | 0.12 | 1.42 | 0.92 |
| 0.03 mg/kg | Primary bile acid biosynthesis | C00037 | Glycine | -0.34 | 0.04 | 0.53 | 1.72 | 0.83 |
| 0.03 mg/kg | Primary bile acid biosynthesis | C05122 | Taurocholate | -0.48 | 0.03 | 0.13 | 1.34 | 0.81 |
| 0.03 mg/kg | Primary bile acid biosynthesis | C01301 | 3a,7a,12a-Trihydroxy-5b-cholestan-26-al | -0.72 | 0.02 | 0.12 | 1.41 | 0.81 |
| 0.03 mg/kg | Primary bile acid biosynthesis | C17337 | 7 alpha-Hydroxy-3-oxo-4-cholestenoate | -1.08 | 0.01 | 0.09 | 1.53 | 0.94 |
| 0.03 mg/kg | Primary bile acid biosynthesis | C05122 | Taurocholic acid | -1.11 | 0.001 | 0.06 | 1.73 | 0.84 |
| 0.03 mg/kg | Primary bile acid biosynthesis | C05452 | 3a,7a-Dihydroxy-5b-cholestane | -0.34 | 0.01 | 0.11 | 1.35 | 0.22 |
| 0.03 mg/kg | Primary bile acid biosynthesis | C00695 | Cholic acid | 1.57 | 0.04 | 0.52 | 1.84 | 0.80 |
| 0.03 mg/kg | Primary bile acid biosynthesis | C00245 | Taurine | -0.86 | 0.03 | 0.51 | 1.81 | 0.83 |
| 0.03 mg/kg | Glycerophospholipid metabolism | 313977 | Lpin1 | -0.73 | 0.03 | 1.00 | - | 0.88 |
| 0.03 mg/kg | Glycerophospholipid metabolism | 298579 | Pla2g2d | -1.27 | 0.01 | 1.00 | - | 0.94 |
| 0.03 mg/kg | Porphyrin and chlorophyll metabolism | 289533 | Ugt2a3 | 0.96 | 0.02 | 1.00 | - | 0.12 |
| 0.03 mg/kg | Porphyrin and chlorophyll metabolism | 65155 | Alas1 | 1.07 | 0.002 | 0.57 | - | 0.00 |
| 0.03 mg/kg | Sphingolipid metabolism | 313339 | Acer2 | -0.64 | 0.02 | 1.00 | - | 1.00 |
| 0.03 mg/kg | Primary bile acid biosynthesis | 25428 | Cyp7a1 | -1.16 | 0.01 | 1.00 | - | 0.88 |
| 0.3 mg/kg | Retinol metabolism | C00899 | 11-cis-Retinol | -0.48 | 0.04 | 0.55 | 1.68 | 0.8 |
| 0.3 mg/kg | Linoleic acid metabolism | C14826 | 12,13-EpOME | -0.69 | 0.01 | 0.36 | 2.11 | 0.86 |
| 0.3 mg/kg | Linoleic acid metabolism | C00157 | PC(18:4(6Z,9Z,12Z,15Z)/P-16:0) | -1.19 | 0.02 | 0.49 | 1.89 | 0.97 |
| 0.3 mg/kg | Linoleic acid metabolism | C00157 | PC(18:0/22:6(4Z,7Z,10Z,13Z,16Z,19Z)) | 1.02 | 0.04 | 0.49 | 1.67 | 0.19 |
| 0.3 mg/kg | D-Glutamine and D-glutamate metabolism | C02237 | D-Pyroglutamic acid | -0.75 | 0.004 | 0.33 | 2.20 | 0.92 |
| 0.3 mg/kg | D-Glutamine and D-glutamate metabolism | C00026 | alpha-ketoglutarate | 0.51 | 0.02 | 0.49 | 1.85 | 0.14 |
| 0.3 mg/kg | Fatty acid elongation | C01944 | Octanoyl-CoA | 1.39 | 0.01 | 0.47 | 2.05 | 0.12 |
| 0.3 mg/kg | Retinol metabolism | 289533 | Ugt2a3 | 1.42 | 0.002 | 0.58 | - | 0.06 |
| 0.3 mg/kg | Retinol metabolism | 50549 | Cyp4a1 | -1.42 | 0.0003 | 0.21 | - | 1.00 |
| 0.3 mg/kg | Retinol metabolism | 312495 | Cyp26b1 | 1.81 | 0.01 | 0.88 | - | 0.12 |
| 0.3 mg/kg | Retinol metabolism | 24188 | Aldh1a1 | -1.03 | 0.01 | 1.00 | - | 0.88 |
| 0.3 mg/kg | Retinol metabolism | 24296 | Cyp1a1 | 2.35 | 0.003 | 0.59 | - | 0.25 |
| 0.3 mg/kg | Retinol metabolism | 308445 | Cyp2s1 | -2.00 | 0.0002 | 0.18 | - | 0.94 |
| 0.3 mg/kg | Neomycin, kanamycin and gentamicin biosynthesis | 25060 | Hk3 | -1.11 | 0.02 | 1.00 | - | 0.88 |
| 0.3 mg/kg | Neomycin, kanamycin and gentamicin biosynthesis | 24385 | Gck | 4.47 | 0.000003 | 0.01 | - | 0.06 |
| 0.3 mg/kg | Linoleic acid metabolism | 298579 | Pla2g2d | -1.09 | 0.03 | 1.00 | - | 0.88 |
| 0.3 mg/kg | Fatty acid elongation | 192272 | Acot2 | -1.37 | 0.01 | 0.80 | - | 0.75 |
| 0.3 mg/kg | Fatty acid elongation | 498728 | Elovl2 | -1.11 | 0.01 | 1.00 | - | 0.75 |
| 0.3 mg/kg | Fatty acid elongation | 681337 | Acot4 | -1.14 | 0.01 | 1.00 | - | 0.75 |
| 0.3 mg/kg | Fatty acid elongation | 50559 | Acot1 | -1.99 | 0.05 | 1.00 | - | 0.75 |

Notes: Group difference of DEGs and DEMs involving in the significant co-enriched pathways (three in 0.03 mg/kg and four in 0.3 mg/kg) were listed.


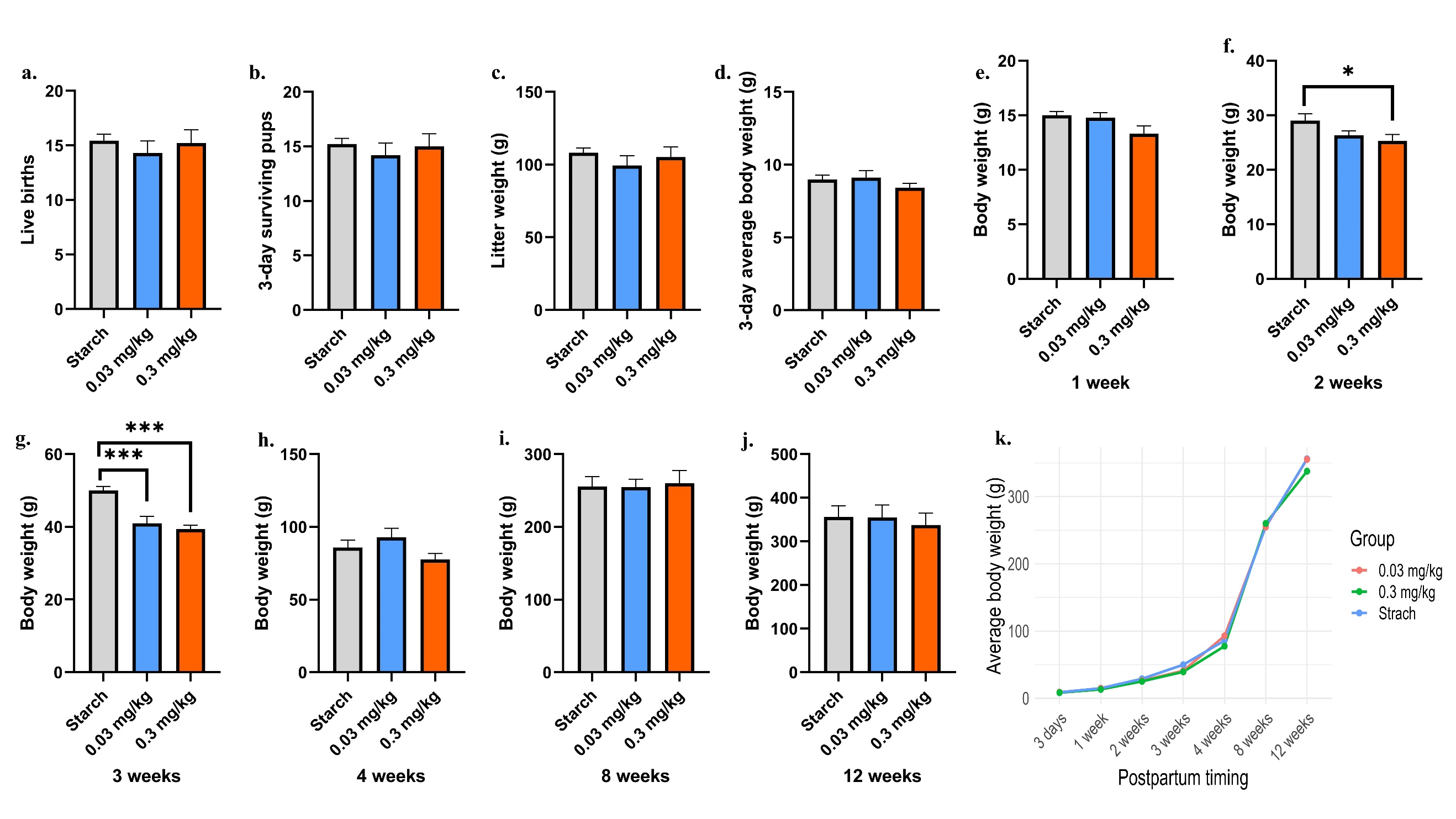


**Fig S1. Effects of early-life PFOS exposure on body weight of offspring in different life stages of rats**. Mean and standard error were reported in broken line plot and bar plot. The sample size of control, PFOS treatment at doses of 0.03 and 0.3 mg/kg groups were 10, 10 and 10, respectively. Two faced *p*-value< 0.05 wet set for significance cutoff. The ratio of male: female offspring rats in each group is same.


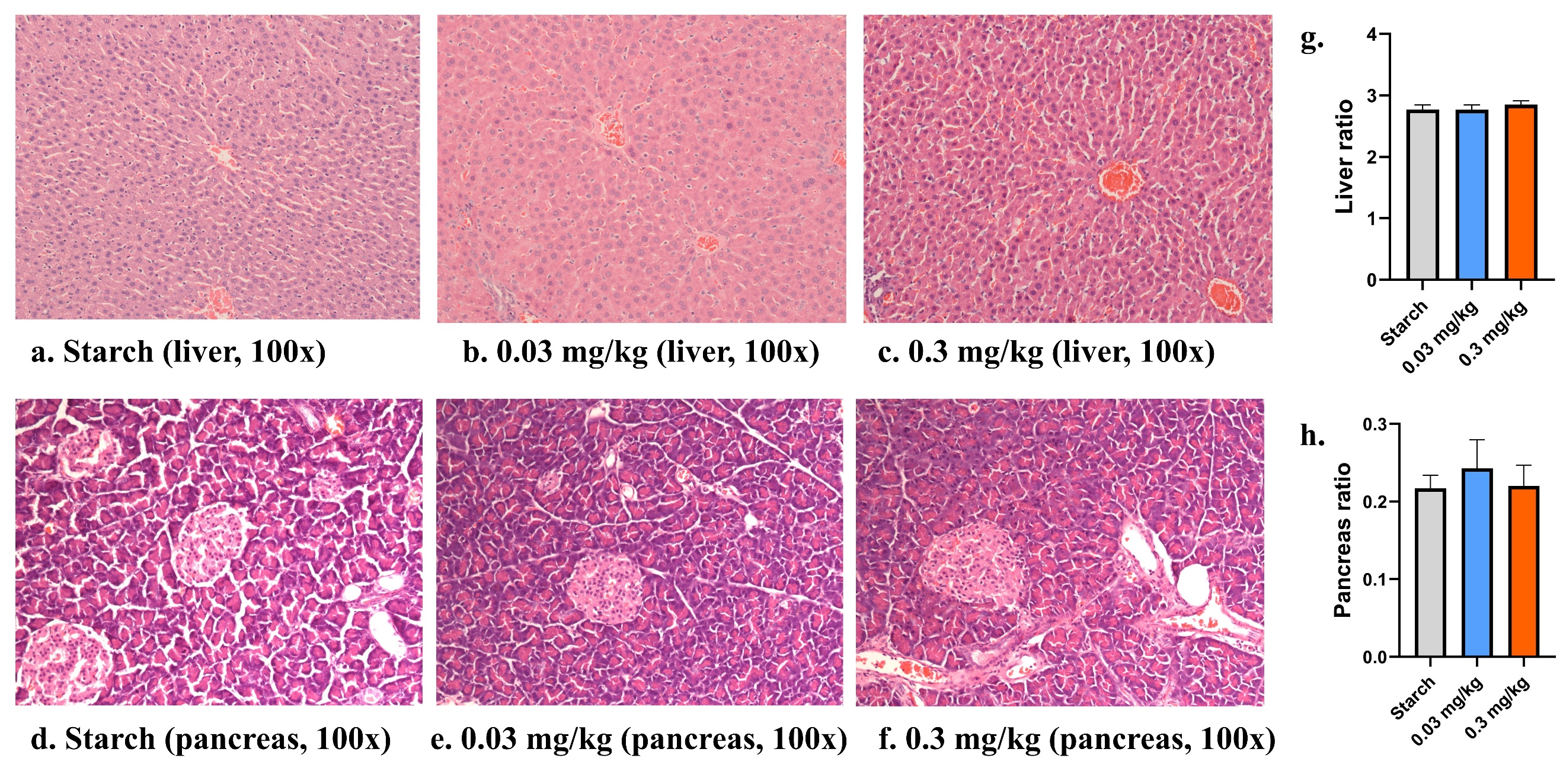


**Fig S2. Pathological changes in liver and pancreas, and their relative weight ratios in 9-week-old offspring rats.** Pathological changes were assessed by hematoxylin and eosin staining. Liver, pancreas and uterus ratio were derived (organ weight/body weight*100). Mean and standard error were reported in bar plot and two faced *p*-value< 0.05 was set as statistically significant.


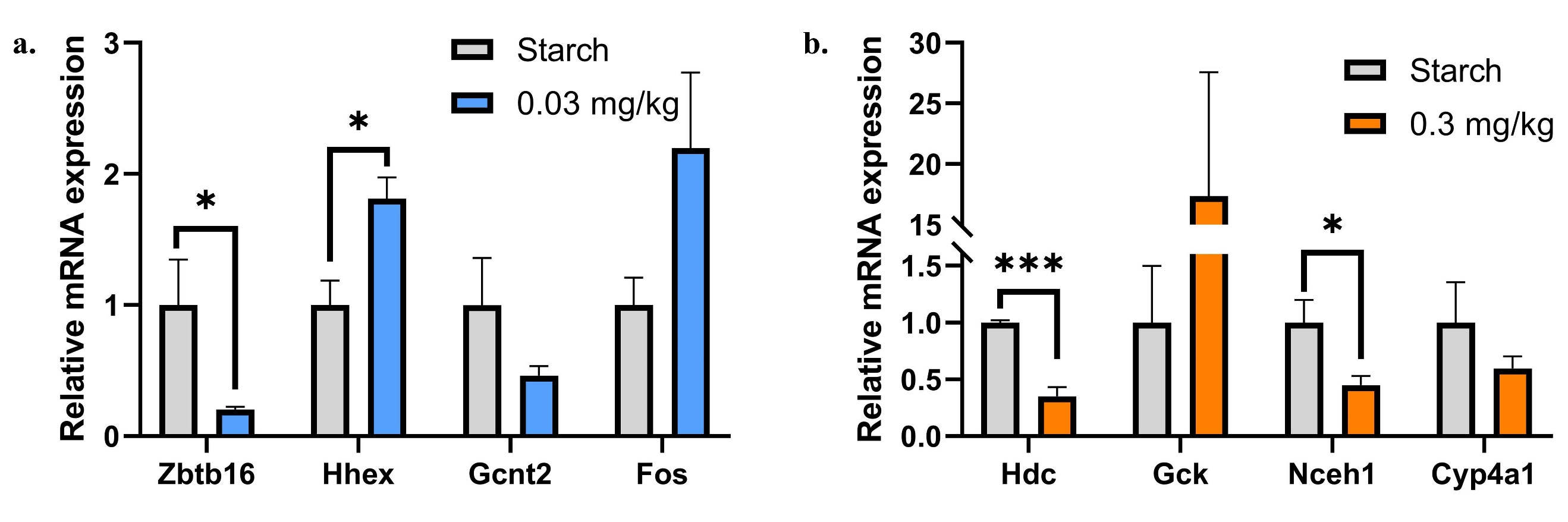


**Fig S3. qPCR validation for the RNAseq transcriptome in liver of 9-week-old offspring rats (N=4).** Mean and standard error were reported. Relative expression level of each selected gene was tested using nonparametric test, with *p*-value< 0.05 as statistically significant. *, *p* < 0.05; **, *p* < 0.01.


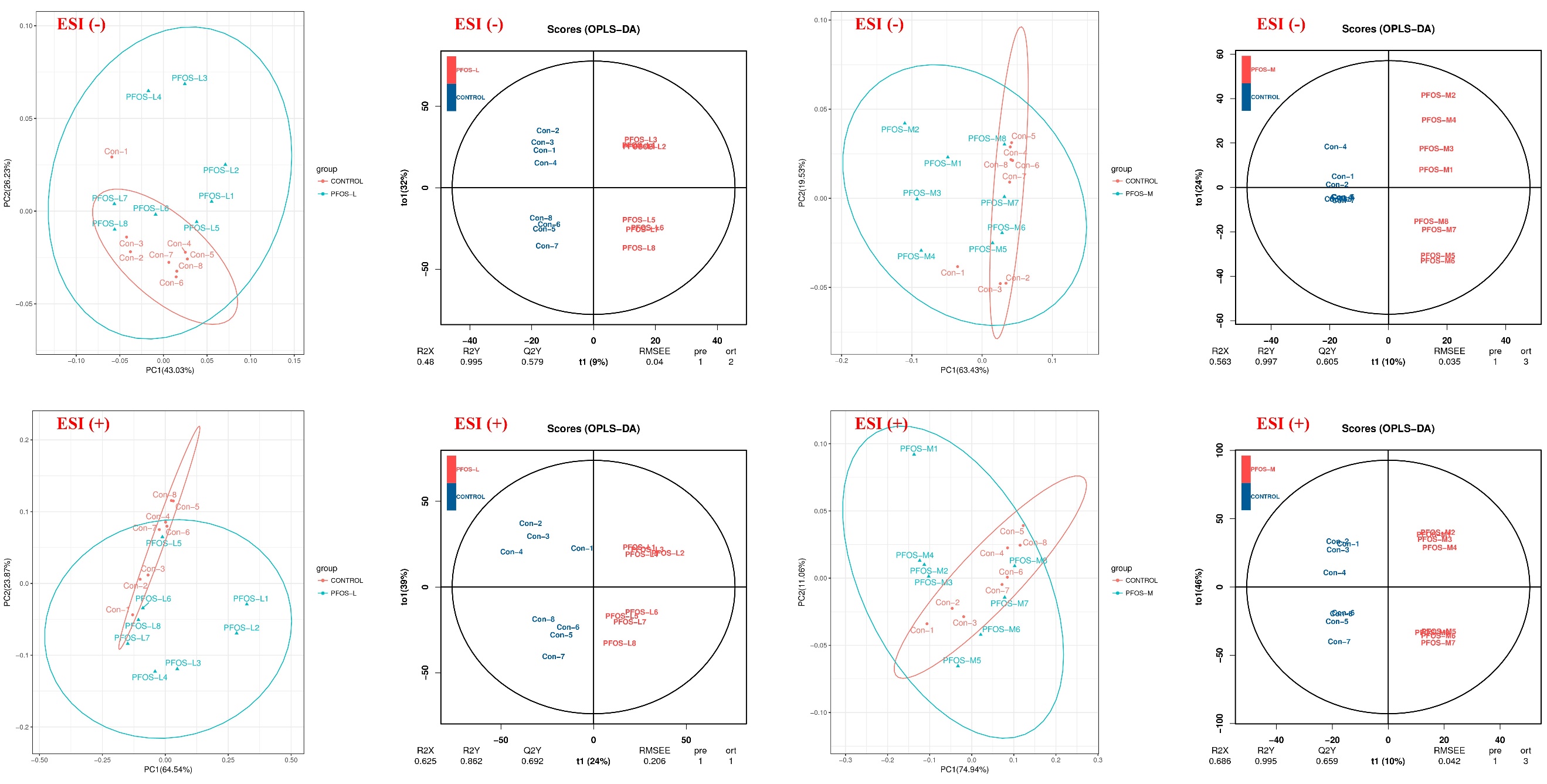


**Fig S4. Principle component analysis and orthogonal projections to latent structures- discriminant analysis for metabolite identification in different ion modes.**


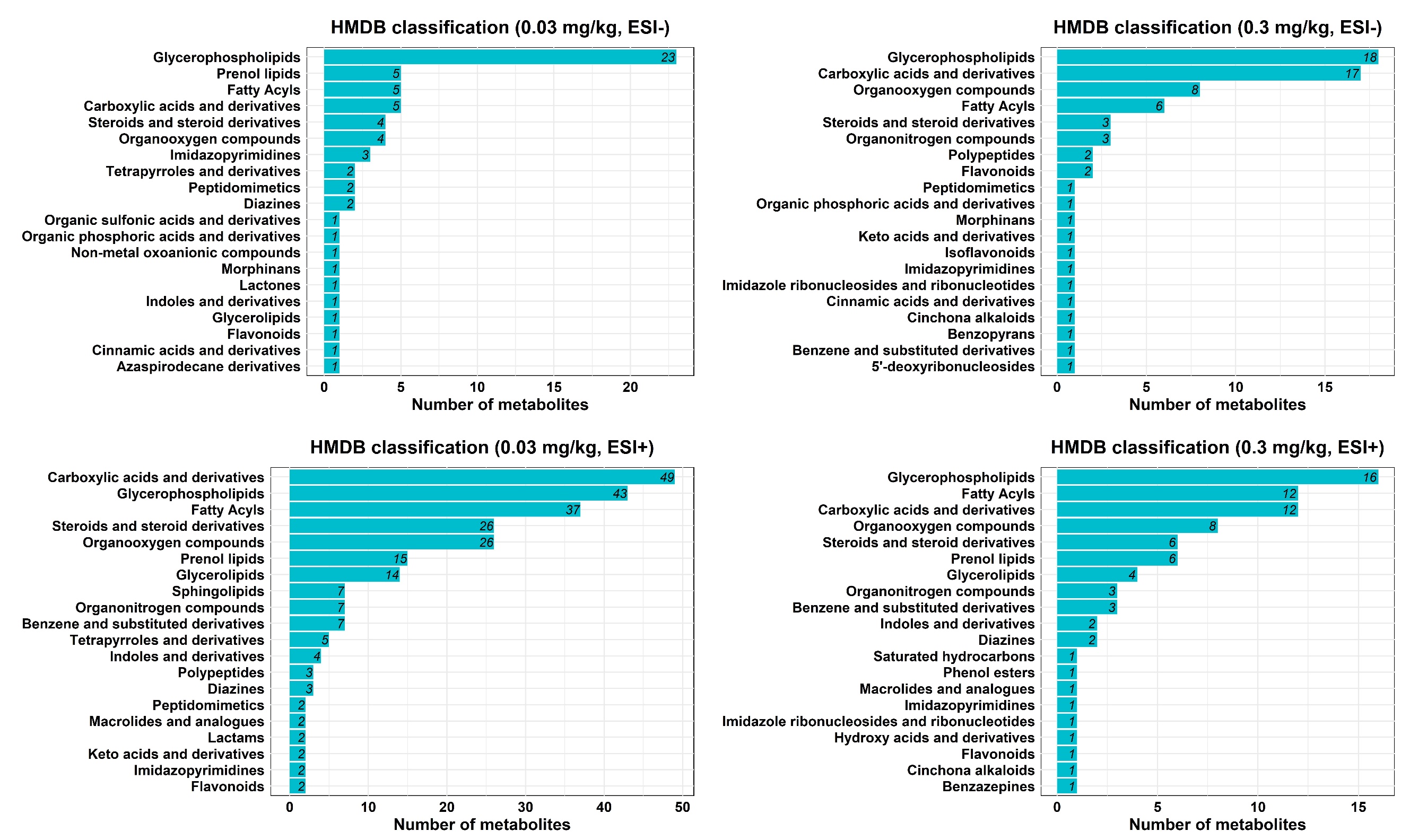


**Fig S5.** **HMDB classification of DEMs** **(N=8/group) in 9-week-old offspring rats.** The width of each bar refers to the number of DEMs in this classification. The top 20 HMDB classifications with highest number of metabolites were visualized.
